# Goal-directed therapy guided by the FloTrac sensor in major surgery: a systematic review and meta-analysis

**DOI:** 10.62675/2965-2774.20240196-en

**Published:** 2024-04-29

**Authors:** Márcia Regina Dias Alves, Saulo Fernandes Saturnino, Ana Beatriz Zen, Dayane Gabriele Silveira de Albuquerque, Henrique Diegoli

**Affiliations:** 1 Edwards Lifesciences São Paulo SP Brazil Edwards Lifesciences - São Paulo (SP), Brazil.; 2 Universidade Federal de Minas Gerais Belo Horizonte MG Brazil Universidade Federal de Minas Gerais - Belo Horizonte (MG), Brazil.; 3 Academia VBHC Educação e Consultoria São Paulo SP Brazil Academia VBHC Educação e Consultoria - São Paulo (SP), Brazil.

**Keywords:** Goals, Monitoring, intraoperative, Length of stay, Heart failure, Treatment outcome, Intensive care units

## Abstract

**Objective:**

To provide insights into the potential benefits of goal-directed therapy guided by FloTrac in reducing postoperative complications and improving outcomes.

**Methods:**

We performed a systematic review and meta-analysis of randomized controlled trials to evaluate goal-directed therapy guided by FloTrac in major surgery, comparing goal-directed therapy with usual care or invasive monitoring in cardiac and noncardiac surgery subgroups. The quality of the articles and evidence were evaluated with a risk of bias tool and GRADE.

**Results:**

We included 29 randomized controlled trials with 3,468 patients. Goal-directed therapy significantly reduced the duration of hospital stay (mean difference -1.43 days; 95%CI 2.07 to -0.79; I^2^ 81%), intensive care unit stay (mean difference -0.77 days; 95%CI -1.18 to -0.36; I^2^ 93%), and mechanical ventilation (mean difference -2.48 hours, 95%CI -4.10 to -0.86, I^2^ 63%). There was no statistically significant difference in mortality, myocardial infarction, acute kidney injury or hypotension, but goal-directed therapy significantly reduced the risk of heart failure or pulmonary edema (RR 0.46; 95%CI 0.23 - 0.92; I^2^ 0%).

**Conclusion:**

Goal-directed therapy guided by the FloTrac sensor improved clinical outcomes and shortened the length of stay in the hospital and intensive care unit in patients undergoing major surgery. Further research can validate these results using specific protocols and better understand the potential benefits of FloTrac beyond these outcomes.

## INTRODUCTION

Surgical interventions are common medical procedures, but despite advancements in surgical techniques, complications due to an imbalance between oxygen supply and demand remain a significant cause of morbidity and mortality.^(1)^Hypoperfusion resulting from this imbalance can lead to a range of complications, including kidney injury, damage to other vital organs and even death.^(2-4)^The risk of complications may be increased by patient characteristics or the nature of the surgical procedure itself. While volume infusion can be used to address hypoperfusion, it is not harmless. Hypervolemia resulting from overhydration can cause heart and kidney failure, extended mechanical ventilation time, and longer hospital stays.^(5-7)^As such, accurate hemodynamic monitoring is essential for guiding fluid management and avoiding adverse outcomes.

Goal-directed therapy (GDT) is a comprehensive approach that employs a range of hemodynamic variables, such as systolic volume variation (SVV) and pulse pressure variation (PPV). The objective is to tailor fluid administration and other therapeutic interventions to individual patient needs, thereby maintaining adequate organ perfusion and minimizing the risks of hypotension and hypervolemia.^(8,9)^Traditional approaches to hemodynamic monitoring rely on invasive methods, such as intra-arterial blood pressure measurements, central venous pressure monitoring, and pulmonary artery catheterization.^(10-12)^These invasive techniques offer high accuracy but are associated with several limitations, including the risk of infection, complications arising from catheter insertion, and elevated costs.^(13-15)^ On the other hand, minimally invasive methods may offer a safer alternative but often at the expense of accuracy, particularly when dynamic parameters are essential.^(13-15)^

In recent years, advances in minimally invasive hemodynamic monitoring technologies, including the FloTrac sensor, have emerged to bridge this gap.^(16)^FloTrac uses an arterial waveform analysis algorithm to estimate cardiac output and other hemodynamic parameters, offering a promising minimally invasive option that overcomes the risks associated with invasive methods. It has been particularly beneficial in GDT due to its balance of safety and accuracy. However, its limitations include a dependence on a stable arterial waveform and potential inaccuracies under specific clinical conditions, such as arrhythmias.^(16)^

Previous meta-analyses have suggested that GDT protocols can reduce the incidence of postoperative complications, particularly in major abdominal, orthopedic, and neurosurgical procedures.^(8)^However, these analyses have also highlighted significant heterogeneity in the devices and protocols used in various studies, which has limited the generalizability of the results. As such, further investigation is warranted to determine the potential benefits of using FloTrac for GDT in high-risk surgical patients undergoing major surgery.

We conducted a systematic review and meta-analysis to evaluate the clinical outcomes and length of stay of patients in hospitals and intensive care units (ICUs) in which FloTrac for GDT was used as opposed to traditional hemodynamic monitoring approaches in patients undergoing major surgery. Our goal was to provide insights into the potential benefits of GDT guided by FloTrac in reducing postoperative complications and improving outcomes.

## Methods

A systematic review was conducted following the Brazilian Guidelines on Systematic Reviews.^(17)^The findings were reported following the criteria set out by the Preferred Reporting Items for Systematic Reviews and Meta-Analyses (PRISMA) Statement (Table 1S - Supplementary Material).^(18)^

### Inclusion and exclusion criteria

We included studies that investigated cardiac output monitoring with FloTrac/Vigileo or FloTrac/HemoSphere using a GDT protocol compared with either invasive hemodynamic monitoring or no continuous cardiac output monitoring (usual care) (Table 2S - Supplementary Material). Goal-directed therapy is defined as an approach that uses specific physiologic parameters to guide clinical treatment decisions. This may involve the use of fluids, inotropes, or other interventions based on continuous or intermittent monitoring.

We included only studies of adult patients involving major surgery, defined by the Delphi Consensus among European Surgical Association members,^(19)^which includes significant patient comorbidity, key surgical parameters (long operative duration, organ ischemia, blood loss > 1000mL, high vasopressor use), postoperative metabolic stress response, 30-day morbidity > 30%, mortality > 2% or the need for intermediate or intensive care.

We included only randomized controlled trials (RCTs) or systematic reviews of RCTs. The compared outcomes were heart failure or pulmonary edema (primary outcome), acute kidney injury, myocardial infarction, hypotension, mortality, length of hospital stay, length of ICU stay and duration of mechanical ventilation. Clinical outcomes of all severities were included, specifically, myocardial infarction (elevated cardiac biomarkers associated with compatible electrocardiogram changes), heart failure or pulmonary edema (signs of pulmonary fluid overload), acute kidney injury (reduced urine output or increased serum creatinine), and hypotension (systolic blood pressure < 90mmHg or diastolic blood pressure < 60mmHg). Studies without any language restrictions were considered eligible for inclusion. Articles published in the form of abstracts or editorial letters were excluded.

### Article identification, selection, and data extraction

The databases used to search for articles were MEDLINE, Cochrane Central Register of Controlled Trials (CENTRAL), and EMBASE. The search strategy for each database and the number of articles identified are shown in table 3S (Supplementary Material), including every article published until January 2023. Articles identified in the databases were pooled and screened using Rayyan.^(20)^

All the articles were screened by two independent reviewers who read the titles and abstracts, and the articles that were considered for inclusion by at least one author were read in full. For final selection and data extraction, the articles were completely read by two reviewers in parallel, and discrepancies were settled by discussion between the authors. Microsoft Excel 365® was used for registering the extracted data.

### Meta-analyses

To summarize the data, the duration of hospital and ICU stays were recorded in days, and the duration of mechanical ventilation was recorded in hours, with conversion performed as needed. In the absence of mean and standard deviation data, we converted the median and interquartile range to the mean and standard deviation, respectively, by assuming a normal distribution. We used the formula standard deviation = (q3 - q1)/1.35, which has been shown to be a superior approach to data omission.^(21,22)^

A random-effects model was used for the meta-analysis conducted in this review to account for heterogeneity among the included studies. The outcomes are presented as risk ratios (RRs) for dichotomous data and mean differences (MDs) for continuous data, both with 95% confidence intervals (95%CIs). The Mantel‒Haenszel method was employed to estimate the pooled effect size for binary variables, and the inverse variance method was used for continuous variables, with the restricted maximum-likelihood estimator used for the between-study variance (tau^2^). The Q-Profile method was used to calculate the confidence intervals for both tau^2^ and tau.

Additionally, heterogeneity, expressed as I^2^, was calculated using Q statistics (DerSimonian‒Laird estimator), where an I^2^ of 0% indicates no evidence of heterogeneity between studies, and 100% suggests that all variability in effect estimation is attributed to heterogeneity between studies. A cutoff of 40% was used to define high heterogeneity. Additional subgroup analyses were conducted to compare the subgroups of cardiac surgery and noncardiac surgery and studies with control groups of pulmonary artery catheter (PAC) monitoring and usual care (UC) patients. The level of statistical significance adopted was 5%, indicating that results with a p value less than 0.05 were considered statistically significant. The meta-analyses were performed with the software RStudio using the meta, metafor, and forestplot packages.

### Quality and bias assessment

We assessed the risk of bias in the included RCTs using the Cochrane Risk of Bias Tool for Randomized Controlled Trials, version 2.0 (RoB 2.0). Each study was evaluated across seven domains: random sequence generation, allocation concealment, blinding of participants and personnel, blinding of outcome assessment, incomplete outcome data, selective reporting, and other bias. A study receiving a green plus in at least three of these domains was considered to have a low risk of bias. Publication bias was assessed using funnel plots, and Egger’s test was applied to detect possible asymmetry.

The quality of evidence was assessed using the Grading of Recommendations, Assessment, Development and Evaluations (GRADE) approach. GRADE is a widely accepted tool used to assess the certainty of evidence in systematic reviews and meta-analyses. The quality of evidence was evaluated based on five criteria: risk of bias, inconsistency, indirectness, imprecision, and publication bias. The GRADE approach classifies the quality of evidence into four categories—high, moderate, low, and very low—based on the overall assessment of the criteria.

## Results

**Figure 1:**
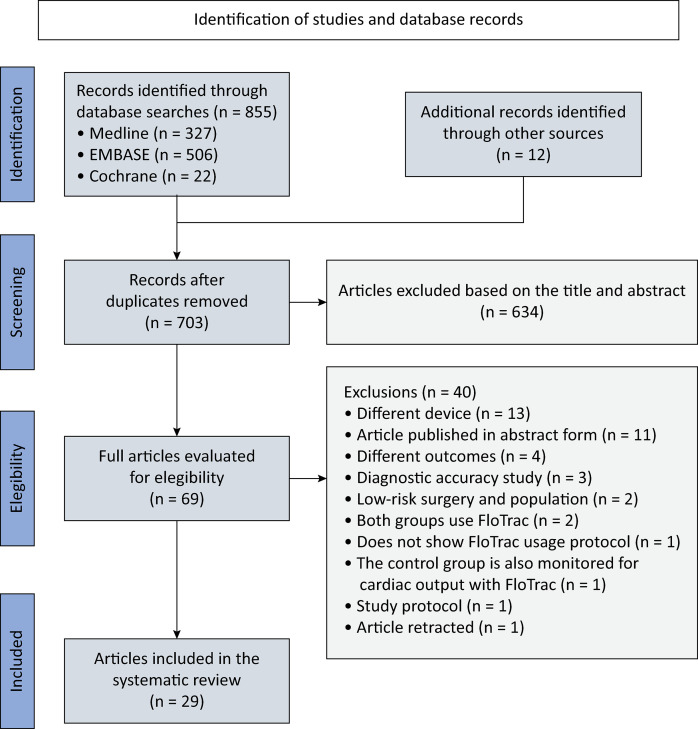
PRISMA flowchart indicating the article inclusion process.

A total of 855 records were initially identified through the database search, with an additional 12 identified through reference checking (Figure 1). After removing duplicates, 703 records remained, and 634 were excluded based on the title and abstract. This left 69 records for full-text review. After a thorough evaluation, 29 articles met all the inclusion and exclusion criteria for the present systematic review. The reasons for excluding articles are presented in table 4S (Supplementary Material).

The present systematic review included 29 RCTs conducted in various countries across five continents, with a total of 1,733 patients in the FloTrac group and 1,735 patients in the control group. Among the included studies, 13 articles included patients who underwent major abdominal surgery (n = 1,671), 8 articles included cardiac surgery patients (n = 1,223), 2 articles included patients who underwent head and neck surgery (n = 200), 2 articles included patients who underwent multiple surgeries (n = 184), 2 articles included patients who underwent major orthopedic surgery (n = 120), 1 article included patients who underwent pulmonary surgery (n = 60), and one article included neurosurgical patients (n = 40). Among these studies, only Hamed et al.^(23)^compared GDT using FloTrac with GDT using an invasive approach (pulmonary artery monitoring), while all other studies compared GDT using FloTrac with usual care, which did not include cardiac output monitoring. Table 1^(16,23-50)^ provides an overview of the main characteristics of the studies included in the review.

**Table 1:** Characteristics of the studies included in the systematic review

Author	Country	Population
Benes et al.^(16)^	Czech Republic	Patients undergoing high-risk major abdominal surgery, or patients with ischemic heart disease, cardiac dysfunction, moderate to severe chronic obstructive pulmonary disease, age above 70 years, or ASA classified as 3 or more
Hamed et al.^(23)^	Egypt	Patients (between 45 and 65 years old) scheduled for saphenous vein graft surgery (2 or more grafts)
Aaen et al.^(24)^	Denmark	Emergency surgery for intestinal obstruction or gastrointestinal perforation in adult patients
Cecconi et al.^(25)^	Italy	Patients undergoing hip arthroplasty surgery
Colantonio et al.^(26)^	Italy	ASA II-III patients diagnosed with peritoneal carcinomatosis candidates for peritonectomy and HIPEC
Gupta et al.^(27)^	India	Patients undergoing resection of head and neck cancer with tissue transfer
Hand et al.^(28)^	United States	Adult patients with tissue transfer surgery for primary reconstruction with head and neck oncological surgeons
Kapoor et al.^(29)^	India	Patients undergoing saphenous vein graft surgery with EuroSCORE greater than or equal to 3
Kapoor et al.^(30)^	India	Patients undergoing saphenous vein graft surgery with EuroSCORE greater than or equal to 3
Kapoor et al.^(31)^	India	Patients undergoing saphenous vein graft surgery with EuroSCORE greater than or equal to 3
Kumar et al.^(32)^	India	Patients undergoing major abdominal surgeries (aortic aneurysm, intestinal obstruction, mesenteric ischemia, intestine cancer involving resection and anastomosis)
Kumar et al.^(33)^	India	ASA I-II patients undergoing major abdominal surgeries (Whipple procedure, resection, retroperitoneal tumor and gastrectomy)
Liu et al.^(34)^	China	ASA II-IV patients over 65 years old undergoing open gastrointestinal surgery (elective)
Martin et al.^(35)^	United Kingdom	Patients between 18 and 80 years old diagnosed with liver cirrhosis and undergoing liver transplantation
Mayer et al.^(36)^	Germany	ASA III patients with 2 or more risk factors undergoing major open abdominal surgery (intestinal/stomach/liver/esophagus resection, Whipple procedure)
Mishra et al.^(37)^	India	ASA I-II patients between 18 and 65 years old with large supratentorial tumors undergoing elective craniotomy and excision
Parke et al.^(38)^	New Zealand	Patients aged 16 or older, classified with EuroSCORE II of 0.9 or more, undergoing elective cardiac surgery
Peng et al.^(39)^	China	Patients undergoing major orthopedic surgeries, including hip arthroplasty, spinal fusion, femur fracture, and sacral tumor
Ramsingh et al.^(40)^	United States	Patients undergoing major abdominal surgeries
Scheeren et al.^(41)^	Germany	ASA III-IV adult patients undergoing high-risk surgery
Sujatha et al.^(42)^	India	ASA I-II patients between 20 and 70 years old undergoing elective intestinal surgery
Tribuddharat et al.^(43)^	Thailand	ASA II-IV adult patients undergoing saphenous vein graft surgery
Tribuddharat et al.^(44)^	Thailand	ASA II-III patients undergoing saphenous vein graft surgery
Van der Linde et al.^(45)^	Belgium	ASA II-III patients undergoing coronary artery bypass surgery
de Waal et al.^(46)^	Netherlands	Patients undergoing high-risk abdominal surgery such as esophagectomy, duodenopancreatectomy, repair of abdominal aneurysm, resection of tissues with malignancy classified above III
Weinberg et al.^(47)^	Australia	Adult patients undergoing major elective liver resection surgery
Zhang et al.^(48)^	China	ASA I-II patients between 18 and 60 years old undergoing thoracoscopic lobectomy surgery under mechanical ventilation of one lung
Zhao et al.^(49)^	China	ASA II-III patients above 60 years old undergoing major abdominal cancer surgery
Zheng et al.^(50)^	China	Patients between 60 and 80 years old with coronary heart disease and undergoing gastrointestinal surgery

In scientific articles where the follow-up time was unclear, we assumed that patients were followed-up until hospital discharge. ASA - American Society of Anesthesiologists Classification; HIPEC - hyperthermic intraperitoneal chemotherapy; UC - usual care; SVV - stroke volume variation; PAC - pulmonary artery catheter.

The quality assessment revealed that 14 of the included articles had a low risk of bias, while 15 had some concerns (Figure 1 - Supplementary Material). The most common causes for concern were deviations from intended interventions (14 articles) and measurement of the outcome (15 articles).

### Synthesis of findings

**Figure 2:**
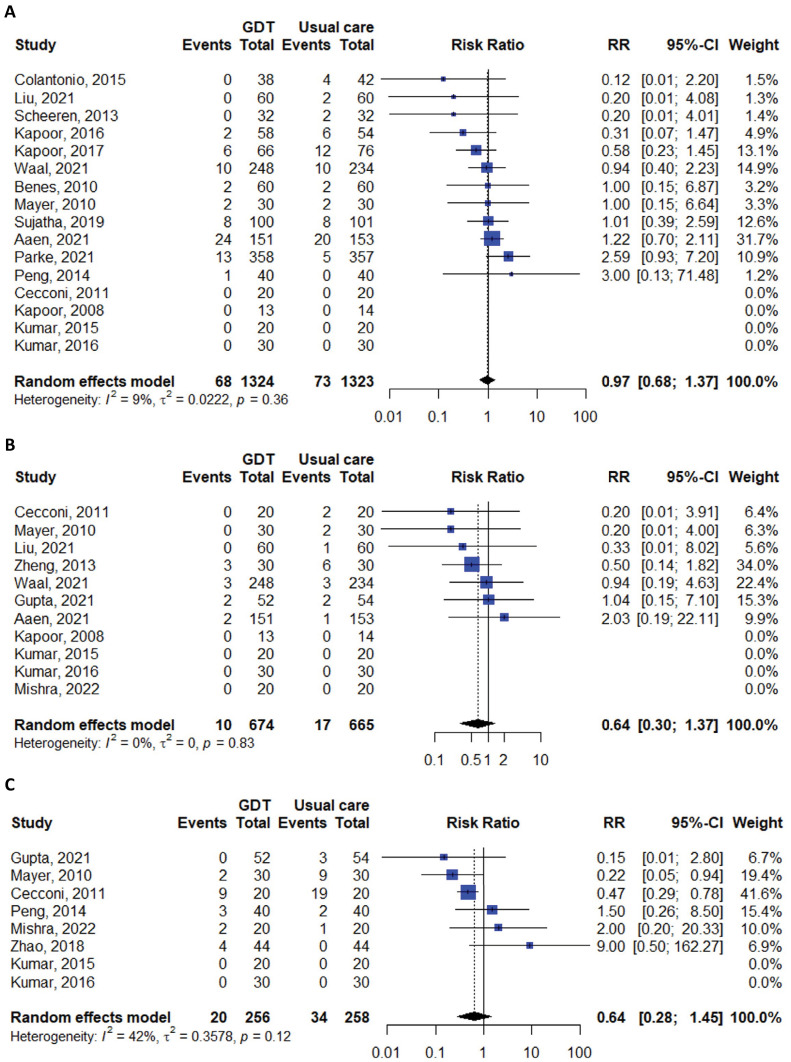
Meta-analyses of studies comparing mortality, myocardial infarction and hypotension between patients receiving goal-directed therapy and those receiving usual care. (A) Mortality; (B) myocardial infarction; (C) hypotension. GDT - goal-directed therapy; RR - risk ratio; 95%CI - 95% confidence interval.

**Figure 3:**
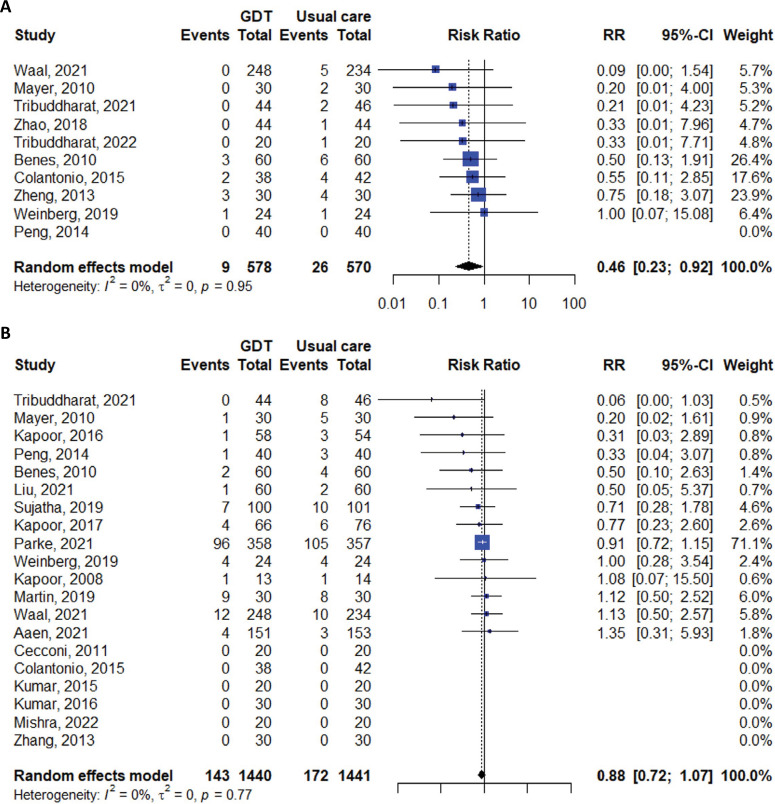
Meta-analyses of studies comparing heart failure or pulmonary edema and acute kidney injury in patients receiving goal-directed therapy *versus* those receiving usual care. (A) Heart failure or pulmonary edema; (B) acute kidney injury. GDT - goal-directed therapy; RR - risk ratio; 95%CI - 95% confidence interval.

The meta-analysis comparing FloTrac to usual care revealed no statistically significant difference between the two groups in terms of mortality (RR 0.97; 95%CI 0.68 - 1.37; I^2^ 9%), myocardial infarction (RR 0.64; 95%CI 0.30 - 1.37; I^2^ 0%), or acute kidney injury (RR 0.88; 95%CI 0.72 - 1.07; I^2^ 0%). However, the risk of heart failure or pulmonary edema was significantly lower in the GDT group (RR 0.46; 95%CI 0.23 - 0.92; I^2^ 0%), while the risk of hypotension was not significantly different (RR 0.64; 95%CI 0.28 - 1.45; I^2^ 42%) (Table 2, Figures 2 and 3).

**Table 2:** Results of the meta-analyses, including subgroup analyses

Outcome - number of studies included	RR or MD (95%CI)	Heterogeneity (I^2^)	Test for subgroup differences (p value)	Test for publication bias (p value)
Mortality - 16 (RR)				
Noncardiac surgery	1.01 (0.69 - 1.49)	0	0.77	0.13
Cardiac surgery	0.84 (0.25 - 2.82)	0.71		
Low risk of bias	1.35 (0.86 - 2.10)	0	0.04	
Some concerns	0.69 (0.42 - 1.11)	0		
Overall	0.97 (0.68 - 1.37)	0.09		
Myocardial infarction - 11 (RR)				
Noncardiac surgery	0.64 (0.30 - 1.37)	0	NA	0.51
Cardiac surgery	NA	NA		
Low risk of bias	0.55 (0.21 - 1.42)	0	0.77	
Some concerns	0.72 (0.42 - 2.14)	0		
Overall	0.64 (0.30 - 1.37)	0		
Heart failure or pulmonary edema - 10 (RR)				
Noncardiac surgery	0.50 (0.24 - 1.02)	0	0.58	0.07
Cardiac surgery	0.26 (0.03 - 2.29)	0		
Low risk of bias	0.51 (0.23 - 1.11)	0	0.63	
Some concerns	0.32 (0.06 - 1.69)	0.17		
Overall	0.46 (0.23 - 0.92)	0		
Acute kidney injury - 20 (RR)				
Noncardiac surgery	0.87 (0.58 - 1.30)	0	0.96	0.04
Cardiac surgery	0.88 (0.70 - 1.11)	0.09		
Low risk of bias	0.88 (0.71 - 1.10)	0	0.87	
Some concerns	0.84 (0.50 - 1.42)	0		
Overall	0.88 (0.72 - 1.07)	0		
Hypotension - 8 (RR)				
Noncardiac surgery	0.64 (0.28 - 1.45)	0.42	NA	0.65
Cardiac surgery	NA	NA		
Low risk of bias	0.46 (0.28 - 0.74)	0	0.25	
Some concerns	1.17 (0.26 - 5.21)	0.56		
Overall	0.64 (0.28 - 1.45)	0.42		
Hospital stay - 23 (MD, days)				
Noncardiac surgery	-1.81 (-2.80 - -0.81)	0.74	0.35	0.07
Cardiac surgery	-1.16 (-1.94 - -0.37)	0.89		
Low risk of bias	-1.19 (-2.04 - -0.34)	0.76	0.39	
Some concerns	-1.79 (-2.86 - -0.73)	0.81		
Overall	-1.43 (-2.07 - -0.79)	0.81		
ICU stay - 18 (MD, days)				
Noncardiac surgery	-0.49 (-0.93 - -0.05)	0.73	0.21	0.22
Cardiac surgery	-1.14 (-1.85 - -0.42)	0.97		
Low risk of bias	-0.60 (-1.17 - -0.03)	0.9	0.46	
Some concerns	-0.92 (-1.53 - -0.31)	0.95		
Overall	-0.77 (-1.18 - -0.36)	0.93		
Duration of mechanical ventilation - 11 (MD, hours)				
Noncardiac surgery	-3.44 (-6.13 - -0.76)	0.44	0.68	<.01
Cardiac surgery	-2.19 (-4.14 - -0.25)	0.70		
Low risk of bias	-2.4 (-5.04 - 0.23)	0.48	0.74	
Some concerns	-3.04 (-5.80 - -0.29)	0.77		
Overall	-2.48 (-4.10 - -0.86)	0.63		

RR - risk ratio; MD - mean difference; 95%CI - 95% confidence interval; NA - not available; ICU - intensive care unit.

**Figure 4:**
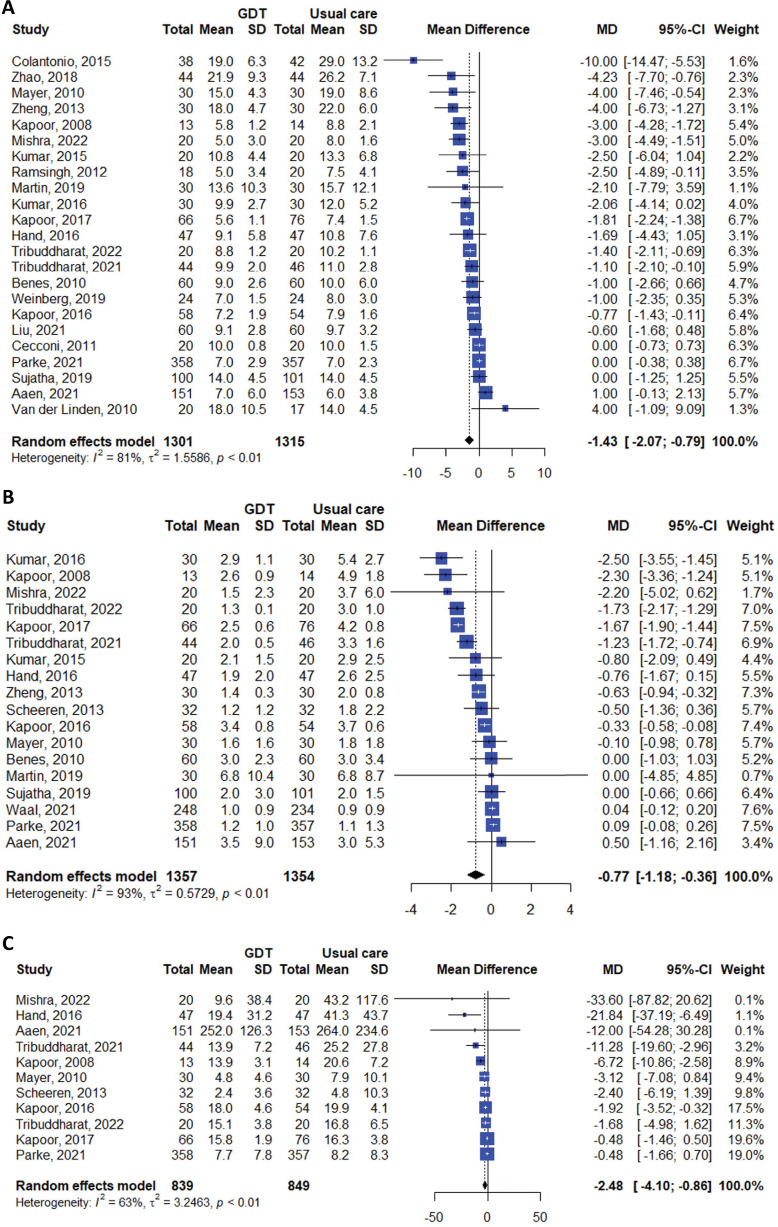
Meta-analyses of studies comparing the length of hospital and intensive care unit stays of patients receiving goal-directed therapy *versus* those receiving usual care. (A) Hospital stay (days); (B) intensive care unit stay (days); (C) duration of mechanical ventilation (hours). GDT - goal-directed therapy; SD - standard deviation; MD - mean difference; 95%CI - 95% confidence interval.

Additionally, the GDT group had a significantly shorter hospital stay (MD -1.43 days; 95%CI -2.07 to -0.79; I^2^ 81%), ICU stay (MD -0.77 days; 95%CI -1.18 to -0.36; I^2^ 93%), and duration of mechanical ventilation (MD -2.48 hours; 95%CI -4.10 to -0.86; I^2^ 68%) (Figure 4).

There was only one study comparing FloTrac with PAC (Hamed et al.,^(23)^). This study reported a reduction in the duration of respiratory support (8.4 *versus* 13.4 hours; p = 0.04) and no statistically significant reduction in the length of ICU stay (55.5 *versus* 58.1 hours; p = 0.7) or length of hospital stay (14.7 *versus* 16.0 days; p = 0.6).

After evaluating the quality of evidence comparing FloTrac and usual care, the results revealed low-quality evidence for acute kidney injury and duration of mechanical ventilation and moderate-quality evidence for the remaining outcomes (Table 3). Funnel plot analysis and Egger’s test showed no signs of publication bias for most outcomes, except for acute kidney injury (p = 0.04) and duration of mechanical ventilation (p < 0.01) (Figure 2S - Supplementary Material).

**Table 3:** Summary of findings table (GRADE)

Outcomes	Illustrative comparison		Relative effect (95%CI)	Number of participants (studies)	Quality of evidence (GRADE)
	Usual care	GDT with FloTrac			
Mortality	55 per 1000	51 per 1000	RR 0.97 (0.68 - 1.37)	2647 (16)	Moderate*
Myocardial infarction	26 per 1000	15 per 1000	RR 0.64 (0.3 - 1.37)	1339 (11)	Moderate*
Hypotension	132 per 1000	78 per 1000	RR 0,64 (0,28 - 1,45)	514 (9)	Moderate*
Heart failure or pulmonary edema	46 per 1000	16 per 1000	RR 0.46 (0.23 - 0.92)	1148 (10)	Moderate*
Acute kidney injury	119 per 1000	99 per 1000	RR 0.88 (0.72 - 1.07)	2881 (20)	Low*†
Hospital stay	10.63 days (ranging between 6 - 29)	-1.43 days (95%CI -2.07 - -0.79)	MD -1.43 (-2.07 - -0.79)	2616 (23)	Moderate‡
ICU stay	2.24 days (ranging between 1 - 7)	-0.77 days (95%CI -1.18 - -0.36)	MD -0.77 (-1.18 - -0.36)	2711 (18)	Moderate‡
Duration of MV	60 hours (ranging between 5 - 264 hours)	-2.48 hours (95%CI -4.10 - -0.86)	MD -2.48 (-4.10 - -0.86)	1688 (11)	Low†‡

GDT - goal-directed therapy; 95%CI - confidence interval; GRADE - Grading of Recommendations, Assessment, Development and Evaluation; RR - risk ratio; MD - mean difference; ICU - intensive care unit; MV - mechanical ventilation; * Imprecision; † the possibility of publication bias; ‡ inconsistency.

Furthermore, when the analysis was stratified by subgroups of cardiac surgery and noncardiac surgery, no statistically significant differences were found between the two groups, suggesting that the type of surgery did not significantly impact the effectiveness of FloTrac compared to the control groups. Subgroup analysis was also conducted to stratify studies based on risk of bias, categorizing them as ‘low risk’ and ‘some concerns.’ Generally, no significant differences in outcomes were observed across these groups. The only exception was in mortality (p = 0.04), but despite the difference between groups, the results in both groups still did not show a statistically significant difference between Flotrac and usual care.

## Discussion

The present systematic review and meta-analysis aimed to assess the effectiveness of GDT guided by the FloTrac sensor compared to traditional approaches for hemodynamic monitoring in reducing postoperative complications and utilizing resources in patients undergoing major surgery. Our findings suggest that GDT guided by the FloTrac sensor may lead to a reduction in the incidence of heart failure or pulmonary edema compared to usual care (54% risk reduction), while there were no statistically significant differences in other clinical outcomes, such as mortality, myocardial infarction, acute kidney injury, or hypotension. Additionally, significant reductions in hospitalization stay (-1.43 days), ICU stay (-0.77 days), and duration of mechanical ventilation (-2.48 hours) were observed, suggesting that the use of GDT protocols, specifically those utilizing the FloTrac sensor, can lead to both improved patient outcomes and more efficient use of health care resources.

It is crucial to understand that the FloTrac sensor itself is not a direct modifier of clinical outcomes. Rather, the actionable insights derived from this minimally invasive hemodynamic monitoring system can empower clinicians to make more informed decisions, potentially leading to improved patient outcomes. Guiding GDT through minimally invasive hemodynamic monitoring allows clinicians to obtain a more precise patient hemodynamic profile using dynamic parameters of fluid responsiveness compared to traditional and static methods.^(11)^This approach allows for appropriate volume infusion, which can avoid the harms of hypo- and hypervolemia and aid in making more accurate decisions regarding inotropic or vasopressor support.^(16)^The results of this study support this rationale, as GDT protocols guided by the FloTrac sensor were found to optimize the choice of appropriate therapy at the correct dose, leading to significant reductions in heart failure or pulmonary edema, hospitalization length of stay, ICU length of stay, and duration of mechanical ventilation. These findings are consistent with previous studies indicating that maintaining perioperative hemodynamic stability can reduce the incidence of postoperative complications.^(4,51-53)^

While our data demonstrate that FloTrac can contribute to a shorter length of stay in both the ICU and hospital settings, the underlying mechanisms are not entirely clear. Although a significant reduction in heart failure or pulmonary edema was observed, FloTrac was not associated with a statistically significant reduction in other postoperative outcomes. Future research should aim to elucidate the specific pathways through which such benefits occur. We also did not focus on other types of complications, such as infections, which could also have an impact on patient outcomes and length of stay.

The incorporation of medical devices into clinical practice requires a rigorous evaluation of the evidence supporting their use. Although, validated instruments exist to assess the accuracy of diagnostic methods,^(54)^incorporating these methods into guided interventions presents a unique challenge for devices, particularly in the context of hemodynamic monitoring.^(55)^The variation in GDT protocols, the learning curve of professionals, and other factors can introduce significant variability into uncontrolled environments. Thus, further validation of specific use protocols may provide further insights into the role of GDT.

Although the present study suggested that GDT protocols guided by FloTrac may optimize the choice of appropriate therapy, it is important to acknowledge some limitations. First, blinding was not possible, as both patients and health care providers are aware of the type of hemodynamic monitoring being used. Additionally, many studies included in the meta-analysis did not report all outcomes sought for the meta-analyses. Moreover, significant differences in patient populations and GDT protocols were observed, increasing the heterogeneity of the analysis.

Another limitation is the inclusion of studies with a 0% mortality rate in the control group. While these studies met other criteria for being categorized as major surgery, the absence of perioperative deaths brings into question the appropriateness of using mortality as an outcome for all surgical populations included in this review. This may be particularly relevant for surgeries with inherently low mortality rates, where other outcomes such as surgical complications or length of hospital or ICU stay might provide a more nuanced understanding of the intervention’s impact.

At the review level, limitations include but are not limited to incomplete retrieval of identified research, missing data for participants or important outcomes, and heterogeneity. The heterogeneity observed in our meta-analysis could be attributed to several factors. Patient populations undergoing major surgery are highly heterogeneous, with variations in baseline characteristics, comorbidities, and surgical procedures, which correlates to the high heterogeneity found in the duration of hospital stay, duration of ICU stay and duration of mechanical ventilation. Additionally, the variations in GDT protocols, such as the criteria, type and timing of fluid administration, may contribute to differences in outcomes. One potential limitation could be the inclusion of studies with different types of control groups, namely, PAC and UC. However, only one study employed PAC as the control. Interestingly, its outcomes did not deviate from the general findings of our review.

Additionally, while most studies had a low risk of bias or only some concerns, many did not provide explicit definitions for key outcomes, such as acute myocardial infarction, acute kidney injury, heart failure, pulmonary edema, and hypotension, and did not indicate the time at which those outcomes occurred (whether intraoperative or postoperative). This lack of standardization could introduce an additional layer of bias in the interpretation. Furthermore, the experience and expertise of health care teams in implementing GDT protocols and interpreting the hemodynamic data provided by the FloTrac sensor may vary across different settings. These limitations highlight the need for further research to explore the clinical utility and optimal implementation of hemodynamic monitoring devices, such as the FloTrac.

## CONCLUSION

This systematic review and meta-analysis suggested that goal-directed therapy protocols guided by the FloTrac sensor lead to improved clinical outcomes and reduced hospital and intensive care unit stays and mechanical ventilation time in patients undergoing major surgery. Further research is needed to validate the results of this study for specific use protocols and to better understand the potential benefits of the FloTrac sensor beyond the outcomes measured in this review. Despite these limitations, the present study provides important insights into the potential benefits of incorporating minimally invasive hemodynamic monitoring into clinical practice and highlights the need for continued research in this area.
