# Supplementary material for: Goal-directed therapy guided by the FloTrac sensor in major surgery: a systematic review and meta-analysis
Source: Crit Care Sci. 2024 Apr 29;36:e20240196en. doi: 10.62675/2965-2774.20240196-en (PMC11098079; doi:10.62675/2965-2774.20240196-en)
Supplement: Supplementary file 1 [file 2965-2774-ccsci-36-e20240196en-suppl01.pdf]

Critical Care Science
https://doi.org/10.62675/2965-2774.20240196-en

SUPPLEMENTARY MATERIAL

Goal-directed therapy guided by the FloTrac sensor in major surgery: a systematic review and meta-analysis

Márcia Regina Dias Alves[1], Saulo Fernandes Saturnino[2], Ana Beatriz Zen[3], Dayane Gabriele Silveira de Albuquerque[1], Henrique Diegoli[3]

Table 1S - PRISMA checklist

Section and Topic	Item #	Checklist item	Reported on page #
TITLE			
Title	1	Identify the report as a systematic review.	1
ABSTRACT			
Abstract	2	See the PRISMA 2020 for Abstracts checklist.	1
INTRODUCTION			
Rationale	3	Describe the rationale for the review in the context of existing knowledge.	2
Objectives	4	Provide an explicit statement of the objective(s) or question(s) the review addresses.	2
METHODS			
Eligibility criteria	5	Specify the inclusion and exclusion criteria for the review and how studies were grouped for the syntheses.	3
Information sources	6	Specify all databases, registers, websites, organisations, reference lists and other sources searched or consulted to identify studies. Specify the date when each source was last searched or consulted.	3; Supplementary table 3
Search strategy	7	Present the full search strategies for all databases, registers and websites, including any filters and limits used.	Supplementary table 3
Selection process	8	Specify the methods used to decide whether a study met the inclusion criteria of the review, including how many reviewers screened each record and each report retrieved, whether they worked independently, and if applicable, details of automation tools used in the process.	3
Data collection process	9	Specify the methods used to collect data from reports, including how many reviewers collected data from each report, whether they worked independently, any processes for obtaining or confirming data from study investigators, and if applicable, details of automation tools used in the process.	4
Data items	10a	List and define all outcomes for which data were sought. Specify whether all results that were compatible with each outcome domain in each study were sought (e.g. for all measures, time points, analyses), and if not, the methods used to decide which results to collect.	4
	10b	List and define all other variables for which data were sought (e.g. participant and intervention characteristics, funding sources). Describe any assumptions made about any missing or unclear information.	3
Study risk of bias assessment	11	Specify the methods used to assess risk of bias in the included studies, including details of the tool(s) used, how many reviewers assessed each study and whether they worked independently, and if applicable, details of automation tools used in the process.	4
Effect measures	12	Specify for each outcome the effect measure(s) (e.g. risk ratio, mean difference) used in the synthesis or presentation of results.	4

Continue...

This is an open access article under the CC BY license (https://creativecommons.org/licenses/by/4.0/).

...continuation

Section and Topic	Item #	Checklist item	Reported on page #
Synthesis methods	13a	Describe the processes used to decide which studies were eligible for each synthesis (e.g. tabulating the study intervention characteristics and comparing against the planned groups for each synthesis (item #5)).	3
	13b	Describe any methods required to prepare the data for presentation or synthesis, such as handling of missing summary statistics, or data conversions.	4
	13c	Describe any methods used to tabulate or visually display results of individual studies and syntheses.	4
	13d	Describe any methods used to synthesize results and provide a rationale for the choice(s). If meta-analysis was performed, describe the model(s), method(s) to identify the presence and extent of statistical heterogeneity, and software package(s) used.	4
	13e	Describe any methods used to explore possible causes of heterogeneity among study results (e.g. subgroup analysis, meta-regression).	4
	13f	Describe any sensitivity analyses conducted to assess robustness of the synthesized results.	4
Reporting bias assessment	14	Describe any methods used to assess risk of bias due to missing results in a synthesis (arising from reporting biases).	4
Certainty assessment	15	Describe any methods used to assess certainty (or confidence) in the body of evidence for an outcome.	4
RESULTS			
Study selection	16a	Describe the results of the search and selection process, from the number of records identified in the search to the number of studies included in the review, ideally using a flow diagram.	5; Figure 1
	16b	Cite studies that might appear to meet the inclusion criteria, but which were excluded, and explain why they were excluded.	Supplementary table 4
Study characteristics	17	Cite each included study and present its characteristics.	Table 1
Risk of bias in studies	18	Present assessments of risk of bias for each included study.	Supplementary figure 1
Results of individual studies	19	For all outcomes, present, for each study: (a) summary statistics for each group (where appropriate) and (b) an effect estimate and its precision (e.g. confidence/credible interval), ideally using structured tables or plots.	Supplementary tables 5-6
Results of syntheses	20a	For each synthesis, briefly summarise the characteristics and risk of bias among contributing studies.	5; Supplementary figure 1
	20b	Present results of all statistical syntheses conducted. If meta-analysis was done, present for each the summary estimate and its precision (e.g. confidence/credible interval) and measures of statistical heterogeneity. If comparing groups, describe the direction of the effect.	6; figures 2-4; tables 2-3; supplementary figure 2
	20c	Present results of all investigations of possible causes of heterogeneity among study results.	-
	20d	Present results of all sensitivity analyses conducted to assess the robustness of the synthesized results.	-
Reporting biases	21	Present assessments of risk of bias due to missing results (arising from reporting biases) for each synthesis assessed.	Supplementary Figure 2
Certainty of evidence	22	Present assessments of certainty (or confidence) in the body of evidence for each outcome assessed.	Figures 2-4
DISCUSSION			
Discussion	23a	Provide a general interpretation of the results in the context of other evidence.	6-8
	23b	Discuss any limitations of the evidence included in the review.	6-8
	23c	Discuss any limitations of the review processes used.	6-8
	23d	Discuss implications of the results for practice, policy, and future research.	8
OTHER INFORMATION			
Registration and protocol	24a	Provide registration information for the review, including register name and registration number, or state that the review was not registered.	-
	24b	Indicate where the review protocol can be accessed, or state that a protocol was not prepared.	-
	24c	Describe and explain any amendments to information provided at registration or in the protocol.	-
Support	25	Describe sources of financial or non-financial support for the review, and the role of the funders or sponsors in the review.	1
Competing interests	26	Declare any competing interests of review authors.	1
Availability of data, code and other materials	27	Report which of the following are publicly available and where they can be found: template data collection forms; data extracted from included studies; data used for all analyses; analytic code; any other materials used in the review.	8

Table 2S - PICOT question used in the systematic review

Category	Description
Population	Adult patients (≥ 18 years) undergoing medium or major surgical procedures or at high surgical risk.
Intervention	Cardiac output monitoring using FloTrac/Vigileo® or FloTrac/HemoSphere® with an utilization protocol
Comparison	Standard hemodynamic monitoring (without continuous cardiac output monitoring)
	Hemodynamic monitoring with Swan-Ganz or other invasive method
Outcome	Myocardial infarction
	Heart failure or pulmonary edema
	Acute kidney injury
	Hypotension
	Length of hospital stay
	Length of intensive care unit stay
Type of study	Randomized controlled trials
	Systematic literature reviews with or without meta-analyses of randomized controlled trials

PICOT - population, intervention, comparison, outcome, time.

Table 3S - Databases searched and search strategy

Database	Search strategy	Articles
MEDLINE	("minimally invasive hemodynamic monitoring" OR "cardiac output monitor" OR "flotrac" OR "vigileo" OR "hemosphere" OR "arterial pressure waveform" OR ("waveform" AND "cardiac output") OR ("minimally invasive" and "goal directed"))<br>AND (("randomized controlled trial"[Publication Type] OR "controlled clinical trial"[Publication Type] OR "randomized"[Title/Abstract] OR "placebo"[Title/Abstract] OR "drug therapy"[MeSH Subheading] OR "randomly"[Title/Abstract] OR "trial"[Title/Abstract] OR "groups"[Title/Abstract]) NOT ("animals"[MeSH Terms] NOT "humans"[MeSH Terms]))	327
EMBASE	('minimally invasive hemodynamic monitoring' OR 'cardiac output monitor' OR 'flotrac' OR 'vigileo' OR 'hemosphere' OR 'arterial pressure waveform' OR ('waveform' AND 'cardiac output') OR ('minimally invasive' AND 'goal directed'))<br>AND ('crossover procedure':de OR 'double-blind procedure':de OR 'randomized controlled trial':de OR 'single-blind procedure':de OR random*:de,ab,ti OR factorial*:de,ab,ti OR crossover*:de,ab,ti OR ((cross NEXT/1 over*):de,ab,ti) OR placebo*:de,ab,ti OR ((doubl* NEAR/1 blind*):de,ab,ti) OR ((singl* NEAR/1 blind*):de,ab,ti) OR assign*:de,ab,ti OR allocat*:de,ab,ti OR volunteer*:de,ab,ti) AND [embase]/lim	506
CENTRAL	#1: (hemodynamic monitoring)<br>#2: (minimally invasive)<br>#3: (cardiac output monitor)<br>#4: #1 AND #2<br>#5: #1 AND #3<br>#6: (flotrac)<br>#7: (vigileo)<br>#8: (hemosphere)<br>#9: #4 OR #5 OR #6 OR #7 OR #8	22

Table 4S - Reasons for article exclusion

Study	Reason for exclusion
Arulkumaran et al.[1]	Article published in abstract form
Arulkumaran et al.[2]	Intervention uses another monitoring model
Ball et al.[3]	Diagnostic accuracy study
Bokhari et al.[4]	Article published in abstract form
Booth et al.[5]	Article published in abstract form
Chatti et al.[6]	Diagnostic accuracy study
Chowdhury et al.[7]	Article published in abstract form
Diaper et al.[8]	Another type of technology used
Donat et al.[9]	Article published in abstract form
Funk et al.[10]	Article does not include any outcomes defined in the methods
Funk et al.[11]	Article does not include any outcomes defined in the methods
Gerent et al.[12]	Article published in abstract form
Gomez-Izquierdo et al.[13]	Article does not include any outcomes defined in the methods
Harten et al.[14]	Uses other devices for cardiac output monitoring
Isosu et al.[15]	Not a randomized clinical trial
Jammer et al.[16]	Uses other devices for cardiac output monitoring
Joosten et al.[17]	The control group also monitors cardiac output with Flotrac
Lai et al.[18]	Intervention uses another monitoring model
Li et al.[19]	Diagnostic accuracy study
Lindroos et al.[20]	Both groups use Flotrac
Lorsomradee et al.[21]	Article published in abstract form
Mayer et al.[22]	Article published in abstract form
Michard et al.[23]	The primary studies of the systematic review include other technologies
Montenij et al.[24]	Study protocol
Mukaiet al.[25]	Article published in abstract form
Murabito et al.[26]	Article does not include any outcomes defined in the methods
Pearse et al.[27]	Intervention uses another monitoring model
Pestaña et al.[28]	Another type of technology used
Ripollés et al.[29]	The primary studies of the systematic review include other technologies
Salzwedel et al.[30]	Intervention uses another monitoring model
Scheeren et al.[31]	Article published in abstract form
Silva Jr et al.[32]	The primary studies of the systematic review include other technologies
Stens et al.[33]	Another type of technology used
Tang et al.[34]	Minimally invasive procedure
Turkut et al.[35]	Low-risk population
van Beest et al.[36]	Both groups use Flotrac
Van der Linden et al.[37]	Article published in abstract form
de Witte et al.[38]	Does not present the FloTrac usage protocol
Yin et al.[39]	Another type of technology used
Zeng et al.[40]	Article retracted

Table 5S - Clinical findings of the studies included in the systematic review

Study	Mortality n (%)		Myocardial infarction n (%)		Heart failure or pulmonary edema n (%)		Acute kidney injury n (%)		Hypotension n (%)	
	Flotrac	Control	Flotrac	Control	Flotrac	Control	Flotrac	Control	Flotrac	Control
Benes et al.[41]	2 (3.3)	2 (3.3)			3 (5.0)	6 (10.0)	2 (3.3)	4 (6.7)		
Hamed et al.[42]										
Aaen et al.[43]	24 (15.9)	20 (13.1)	2 (1.3)	1 (0.7)			4 (2.6)	3 (2.0)		
Cecconi et al.[44]	0 (0.0)	0 (0.0)	0 (0.0)	2 (10.0)			0 (0.0)	0 (0.0)	9 (45.0)	19 (95.0)
Colantonio et al.[45]	0 (0.0)	4 (9.5)			2 (5.3)	4 (9.5)	0 (0.0)	0 (0.0)		
Gupta et al.[46]			2 (3.8)	2 (3.7)					0 (0.0)	3 (5.6)
Hand et al.[47]										
Kapoor et al.[48]	0 (0.0)	0 (0.0)	0 (0.0)	0 (0.0)			1 (7.7)	1 (7.1)		
Kapoor et al.[49]	2 (3.4)	6 (11.1)					1 (1.7)	3 (5.6)		
Kapoor et al.[50]	6 (9.1)	12 (15.8)					4 (6.1)	6 (7.9)		
Kumar et al.[51]	0 (0.0)	0 (0.0)	0 (0.0)	0 (0.0)			0 (0.0)	0 (0.0)	0 (0.0)	0 (0.0)
Kumar et al.[52]	0 (0.0)	0 (0.0)	0 (0.0)	0 (0.0)			0 (0.0)	0 (0.0)	0 (0.0)	0 (0.0)
Liu et al.[53]	0 (0.0)	2 (3.3)	0 (0.0)	1 (1.7)			1 (1.7)	2 (3.3)		
Martin et al.[54]							9 (30.0)	8 (26.7)		
Mayer et al.[55]	2 (6.7)	2 (6.7)	0 (0.0)	2 (6.7)	0 (0.0)	2 (6.7)	1 (3.3)	5 (16.7)	2 (6.7)	9 (30.0)
Mishra et al.[56]			0 (0.0)	0 (0.0)			0 (0.0)	0 (0.0)	2 (10.0)	1 (5.0)
Parke et al.[57]	13 (3.6)	5 (1.4)					96 (26.8)	105 (29.4)		
Peng et al.[58]	1 (2.5)	0 (0.0)			0 (0.0)	0 (0.0)	1 (2.5)	3 (7.5)	3 (7.5)	2 (5.0)
Ramsingh et al.[59]										
Scheeren et al.[60]	0 (0.0)	2 (6.3)								
Sujatha et al.[61]	8 (8.0)	8 (7.9)					7 (7.0)	10 (9.9)		
Tribuddharat et al.[62]					0 (0.0)	2 (4.3)	0 (0.0)	8 (17.4)		
Tribuddharat et al.[63]					0 (0.0)	1 (5.0)				
Van der Linden et al.[64]										
de Waal et al.[65]	10 (4.0)	10 (4.3)	3 (1.2)	3 (1.3)	0 (0.0)	5 (2.1)	12 (4.8)	10 (4.3)		
Weinberg et al.[66]					1 (4.2)	1 (4.2)	4 (16.7)	4 (16.7)		
Zhang et al.[67]							0 (0.0)	0 (0.0)		
Zhao et al.[68]					0 (0.0)	1 (2.3)			4 (9.1)	0 (0.0)
Zheng et al.[69]			3 (10.0)	6 (20.0)	3 (10.0)	4 (13.3)				

Table 6S - Findings related to duration of hospital stay, intensive care unit stay, and duration of mechanical ventilation

Study	Hospital stay (days) Mean (SD)		ICU stay (days) Mean (SD)		Duration of MV (hours) Mean (SD)	
	Flotrac	Control	Flotrac	Control	Flotrac	Control
Benes et al.[41]	9.00 (2.63)	10.00 (6.02)	3.00 (2.26)	3.00 (3.38)		
Hamed et al.[42]	14.70 (7.80)	16.00 (7.80)	2.31 (1.55)	2.42 (1.57)	8.40 (5.04)	13.44 (5.76)
Aaen et al.[43]	7.00 (6.02)	6.00 (3.76)	3.50 (9.02)	3.00 (5.26)	252.00 (126.32)	264.00 (234.59)
Cecconi et al.[44]	10.00 (0.75)	10.00 (1.50)				
Colantonio et al.[45]	19.00 (6.29)	29.00 (13.23)				
Gupta et al.[46]						
Hand et al.[47]	9.11 (5.76)	10.80 (7.65)	1.88 (2.01)	2.64 (2.49)	19.44 (31.20)	41.28 (43.68)
Kapoor et al.[48]	5.80 (1.20)	8.80 (2.10)	2.60 (0.90)	4.90 (1.80)	13.92 (3.12)	20.64 (7.20)
Kapoor et al.[49]	7.17 (1.93)	7.94 (1.64)	3.41 (0.75)	3.74 (0.59)	18.00 (4.56)	19.92 (4.08)
Kapoor et al.[50]	5.61 (1.11)	7.42 (1.48)	2.53 (0.56)	4.20 (0.82)	15.84 (1.92)	16.32 (3.84)
Kumar et al.[51]	10.85 (4.39)	13.35 (6.77)	2.10 (1.52)	2.90 (2.51)		
Kumar et al.[52]	9.90 (2.68)	11.96 (5.15)	2.90 (1.15)	5.40 (2.71)		
Liu et al.[53]	9.10 (2.80)	9.70 (3.20)				
Martin et al.[54]	13.60 (10.30)	15.70 (12.10)	6.80 (10.40)	6.80 (8.70)		
Mayer et al.[55]	15.00 (4.32)	19.00 (8.65)	1.65 (1.65)	1.75 (1.81)	4.80 (4.56)	7.92 (10.08)
Mishra et al.[56]	5.00 (3.00)	8.00 (1.60)	1.50 (2.30)	3.70 (6.00)	9.60 (38.40)	43.20 (117.60)
Parke et al.[57]	7.00 (2.86)	7.00 (2.33)	1.16 (0.99)	1.07 (1.34)	7.68 (7.76)	8.16 (8.30)
Peng et al.[58]						
Ramsingh et al.[59]	5.00 (3.38)	7.50 (4.14)				
Scheeren et al.[60]			1.25 (1.21)	1.75 (2.17)	2.40 (3.60)	4.80 (10.32)
Sujatha et al.[61]	14.00 (4.51)	14.00 (4.51)	2.00 (3.01)	2.00 (1.50)		
Tribuddharat et al.[62]	9.90 (2.00)	11.00 (2.80)	2.04 (0.48)	3.27 (1.64)	13.92 (7.20)	25.20 (27.84)
Tribuddharat et al.[63]	8.80 (1.20)	10.20 (1.10)	1.28 (0.14)	3.01 (0.99)	15.12 (3.84)	16.80 (6.48)
Van der Linden et al.[64]	18.00 (10.53)	14.00 (4.51)				
de Waal et al.[65]			0.96 (0.88)	0.92 (0.88)		
Weinberg et al.[66]	7.00 (1.50)	8.00 (3.01)				
Zhang et al.[67]						
Zhao et al.[68]	21.93 (9.34)	26.16 (7.12)				
Zheng et al.[69]	18.00 (4.70)	22.00 (6.02)	1.35 (0.32)	1.98 (0.80)		

ICU - intensive care unit; SD - standard deviation; MV - mechanical ventilation.

Studi ID	D1	D2	D3	D4	D5	Overall
Aaen 2021	+	+	+	+	+	+
Benes 2010	+	+	+	+	+	+
Cecconi 2011	+	+	+	!	+	!
Colantonio 2015	+	!	+	!	+	!
Gupta 2021	+	+	+	!	+	!
Hamed 2018	+	!	+	!	+	!
Hand 2016	+	!	+	!	+	!
Kapoor 2008	+	!	+	!	+	!
Kapoor 2016	+	!	+	!	+	!
Kapoor 2017	+	!	+	!	+	!
Kumar 2015	+	!	+	!	+	!
Kumar 2016	+	+	+	!	+	!
Liu 2021	+	+	+	+	+	+
Martin 2019	+	+	+	+	+	+
Mayer 2010	+	!	+	+	+	+
Mishra 2022	+	+	+	+	+	+
Parke 2021	+	+	+	+	+	+
Peng 2014	+	+	+	+	+	+
Ramsingh 2012	+	+	+	!	+	!
Scheeren 2013	+	!	+	!	+	!
Sujatha 2019	+	!	+	!	+	!
Tribuddharat 2021	+	+	+	+	+	+
Tribuddharat 2022	+	+	+	+	+	+
Van der Linden 2010	+	+	+	+	+	+
Waal 2021	+	!	+	!	+	!
Weinberg 2019	+	+	+	+	+	+
Zhang 2013	+	!	+	!	+	!
Zhao 2018	+	!	+	+	+	+
Zheng 2013	+	!	+	+	+	+

+ Low risk
! Some concerns
- High risk

D1 Randomisation process
D2 Deviations from the intended interventions
D3 Missing outcome data
D4 Measurement of the outcome
D5 Selection of the reported result

Figure 1S - Assessment of risk of bias.
D - domain.

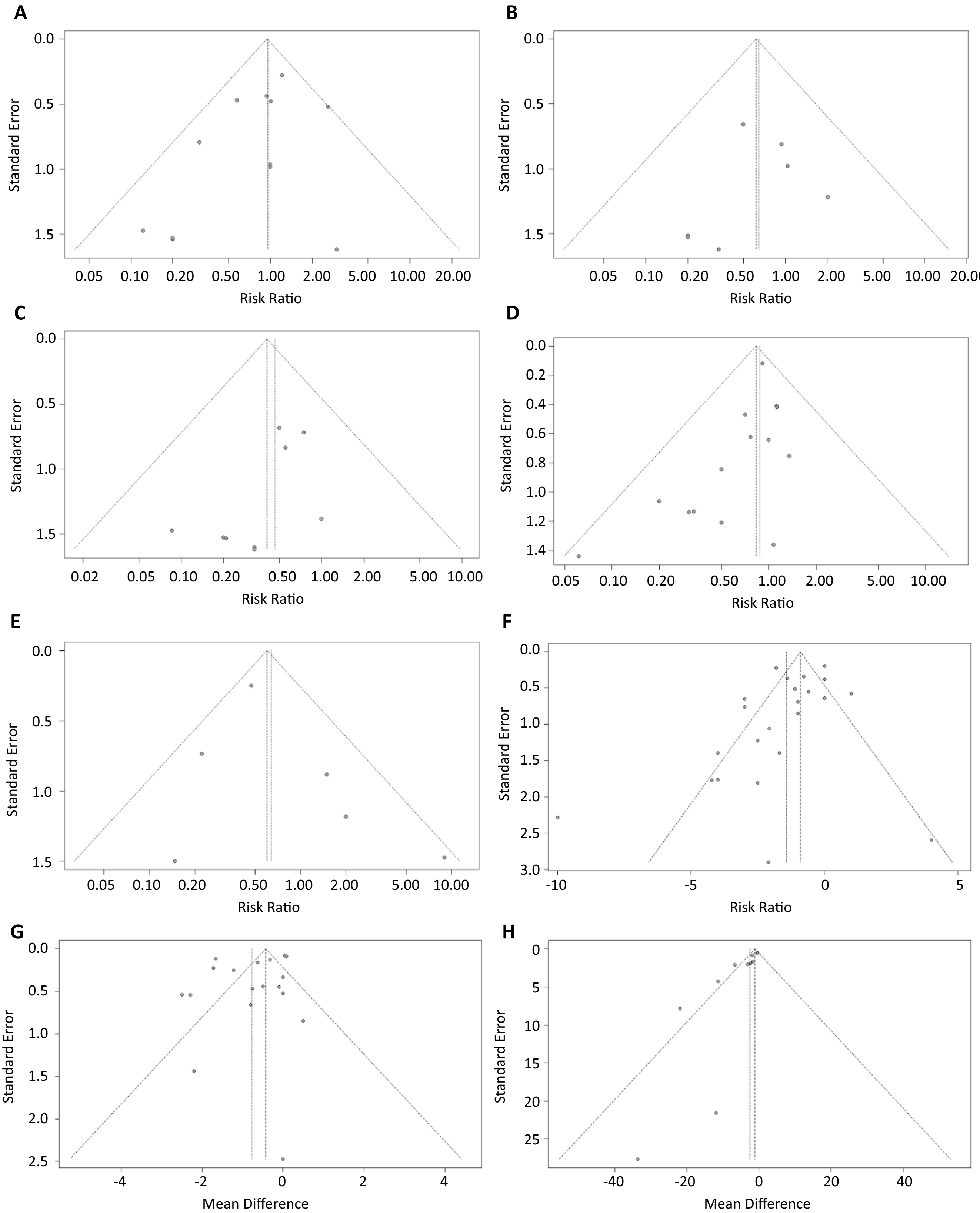

Figure 2S - Funnel plots evaluating publication bias.

(A) mortality; (B) myocardial infarction; (C) acute kidney injury; (D) heart failure; (E) hypotension; (F) hospital stay; (G) ICU stay; (H) duration of mechanical ventilation.

REFERENCES

1. Arulkumaran N, Corredor C, Hamilton M, Grounds M, Ball J, Rhodes A, et al. Treatment-related cardiac complications associated with goal-directed therapy in high-risk surgical patients: a meta-analysis. Crit Care. 2013;17(Suppl 2):P195.
2. Arulkumaran N, Corredor C, Hamilton MA, Ball J, Grounds RM, Rhodes A, et al. Cardiac complications associated with goal-directed therapy in high-risk surgical patients: a meta-analysis. Br J Anaesth. 2014;112(4):648-59.
3. Ball TR, Culp BC, Patel V, Gloyna DF, Ciceri DP, Culp WC Jr. Comparison of the endotracheal cardiac output monitor to thermodilution in cardiac surgery patients. J Cardiothorac Vasc Anesth. 2010;24(5):762-6.
4. Bokhari R, You EL, Lasty O, Baldini G, Weber M. Effect of peri-operative goal-directed fluid therapy on orthopaedic surgery outcomes: a systematic review. Global Spine J. 2018;8(1 Suppl):A319.
5. Booth JH, Quinn TA, Richmond ME, Cabreriza SE, Weinberg AD, Johnston T, et al. Cardiac output measurement by arterial pressure waveform analysis during optimization of biventricular pacing after cardiac surgery. ASAIO J. 2009;55(6):587-91.
6. Chatti R, de Rudniki S, Marqué S, Dumenil AS, Descorps-Declère A, Cariou A, et al. Comparison of two versions of the Vigileo-FloTrac system (1.03 and 1.07) for stroke volume estimation: a multicentre, blinded comparison with oesophageal Doppler measurements. Br J Anaesth. 2009;102(4):463-9.
7. Chowdhury I, Ahuja P. Impact of goal directed intraoperative fluid administration on blood lactate levels and thromboelastography in major oncosurgery patients. Anesth Analg. 2021;133(3 Suppl 2):930.
8. Diaper J, Schiffer E, Barcelos GK, Luise S, Schorer R, Ellenberger C, et al. Goal-directed hemodynamic therapy versus restrictive normovolemic therapy in major open abdominal surgery: a randomized controlled trial. Surgery. 2021;169(5):1164-74.
9. Donat SM, Tan KS, Dalbagni G, Pedoto AC, Herr HW, Bochner BH, et al. PD55-01 a prospective single-blinded randomized controlled clinical trial of perioperative goal-directed fluid therapy versus standard fluid therapy for patients undergoing open radical cystectomy on a standardized postoperative enhanced recovery pathway. J Urol. 2020;203:e1182.
10. Funk DJ, HayGlass KT, Koulack J, Harding G, Boyd A, Brinkman R. A randomized controlled trial on the effects of goal-directed therapy on the inflammatory response open abdominal aortic aneurysm repair. Crit Care. 2015;19(1):247.
11. Funk D, Bohn J, Mutch W, Hayakawa T, Buchel EW. Goal-directed fluid therapy for microvascular free flap reconstruction following mastectomy: a pilot study. Plast Surg (Oakv). 2015;23(4):231-4.
12. Gerent A, Almeida JP, Galas F, Fukushima JT, Osawa E, Park C, et al. Goal-directed therapy in cancer surgery: a randomised and controlled trial (GRICS II). Intensive Care Med Exp. 2015;3(Suppl 1):A819.
13. Gómez-Izquierdo JC, Trainito A, Mirzakandov D, Stein BL, Liberman S, Charlebois P, et al. Goal-directed fluid therapy does not reduce primary postoperative ileus after elective laparoscopic colorectal surgery: a randomized controlled trial. Anesthesiology. 2017;127(1):36-49.
14. Harten J, Crozier JE, McCreath B, Hay A, McMillan DC, McArdle CS, et al. Effect of intraoperative fluid optimisation on renal function in patients undergoing emergency abdominal surgery: a randomised controlled pilot study (ISRCTN 11799696). Int J Surg. 2008;6(3):197-204.
15. Isosu T, Obara S, Ohashi S, Hosono A, Nakano Y, Imaizumi T, Et Al. Examination of the usefulness of non-invasive stroke volume variation monitoring for adjusting fluid supplementation during laparoscopic adrenalectomy in patients with pheochromocytoma. Fukushima J Med Sci. 2012;58(1):78-81.
16. Jammer I, Tuovila M, Ulvik A. Stroke volume variation to guide fluid therapy: is it suitable for high-risk surgical patients? A terminated randomized controlled trial. Perioper Med (Lond). 2015;4:6.
17. Joosten A, Rinehart J, Van der Linden P, Alexander B, Penna C, De Montblanc J, et al. Computer-assisted individualized hemodynamic management reduces intraoperative hypotension in intermediate- and high-risk surgery: a randomized controlled trial. Anesthesiology. 2021;135(2):258-72.
18. Lai CW, Starkie T, Creanor S, Struthers RA, Portch D, Erasmus PD, et al. Randomized controlled trial of stroke volume optimization during elective major abdominal surgery in patients stratified by aerobic fitness. Br J Anaesth. 2015;115(4):578-89.
19. Li C, Lin FQ, Fu SK, Chen GQ, Yang XH, Zhu CY, et al. Stroke volume variation for prediction of fluid responsiveness in patients undergoing gastrointestinal surgery. Int J Med Sci. 2013;10(2):148-55.
20. Lindroos AC, Niiya T, Randell T, Niemi TT. Stroke volume-directed administration of hydroxyethyl starch (HES 130/0.4) and Ringer's acetate in prone position during neurosurgery: a randomized controlled trial. J Anesth. 2014;28(2):189-97.
21. Lorsomradee S, Lorsomradee S, Kitswat P. A comparison of return of gastrointestinal function between perioperative goal-directed therapy and traditional fluid therapy in major abdominal surgery patients: a prospective randomized controlled study. Anaesth Intensive Care. 2017;45(5):641.
22. Mayer J, Boldt J, Beschmann R, Stephan A, Suttner S. Individualized intraoperative patient optimization using uncalibrated arterial pressure waveform analysis in high-risk patients undergoing major abdominal surgery. Crit Care. 2009;13(Suppl 1):P219.
23. Michard F, Giglio MT, Brienza N. Perioperative goal-directed therapy with uncalibrated pulse contour methods: impact on fluid management and postoperative outcome. Br J Anaesth. 2017;119(1):22-30.
24. Montenij L, de Waal E, Frank M, van Beest P, de Wit A, Kruitwagen C, et al. Influence of early goal-directed therapy using arterial waveform analysis on major complications after high-risk abdominal surgery: study protocol for a multicenter randomized controlled superiority trial. Trials. 2014;15:360.
25. Mukai A, Suehiro K, Watanabe R, Juri T, Hayashi Y, Tanaka K, et al. Impact of intraoperative goal-directed fluid therapy on major morbidity and mortality after transthoracic oesophagectomy: a multicentre, randomised controlled trial. Br J Anaesth. 2020;125(6):953-61.
26. Murabito P, Astuto M, Sanfilippo F, La Via L, Vasile F, Basile F, et al. Proactive management of intraoperative hypotension reduces biomarkers of organ injury and oxidative stress during elective non-cardiac surgery: a pilot randomized controlled trial. J Clin Med. 2022;11(2):392.
27. Pearse RM, Harrison DA, MacDonald N, Gillies MA, Blunt M, Ackland G, Grocott MP, Ahern A, Griggs K, Scott R, Hinds C, Rowan K; OPTIMISE Study Group. Effect of a perioperative, cardiac output-guided hemodynamic therapy algorithm on outcomes following major gastrointestinal surgery: a randomized clinical trial and systematic review. JAMA. 2014;311(21):2181-90.
28. Pestaña D, Espinosa E, Eden A, Nájera D, Collar L, Aldecoa C, et al. Perioperative goal-directed hemodynamic optimization using noninvasive cardiac output monitoring in major abdominal surgery: a prospective, randomized, multicenter, pragmatic trial: POEMAS Study (PeriOperative goal-directed thErapy in Major Abdominal Surgery). Anesth Analg. 2014;119(3):579-87.
29. Ripollés J, Espinosa A, Martínez-Hurtado E, Abad-Gurumeta A, Casans-Francés R, Fernández-Pérez C, et al. Perioperative goal-directed hemodynamic therapy in noncardiac surgery: a systematic review and meta-analysis. Rev Bras Anestesiol. 2016;66(5):513-28.
30. Salzwedel C, Puig J, Carstens A, Bein B, Molnar Z, Kiss K, et al. Perioperative goal-directed hemodynamic therapy based on radial arterial pulse pressure variation and continuous cardiac index trending reduces postoperative complications after major abdominal surgery: a multi-center, prospective, randomized study. Crit Care. 2013;17(5):R191.
31. Scheeren TW, Wiesenack C, Gerlach H, Marx G. Goal-directed fluid management based on stroke volume variation and stroke volume optimization during high-risk surgery: a pilot multicentre randomized controlled trial. Crit Care. 2011;15(Suppl 1):P36.
32. Silva-Jr JM, Menezes PF, Lobo SM, de Carvalho FH, de Oliveira MA, Cardoso Filho FN, et al. Impact of perioperative hemodynamic optimization therapies in surgical patients: economic study and meta-analysis. BMC Anesthesiol. 2020;20(1):71.

33. Stens J, Hering JP, van der Hoeven CW, Boom A, Traast HS, Garmers LE, et al. The added value of cardiac index and pulse pressure variation monitoring to mean arterial pressure-guided volume therapy in moderate-risk abdominal surgery (COGUIDE): a pragmatic multicentre randomised controlled trial. Anaesthesia. 2017;72(9):1078-87.

34. Tang W, Qiu Y, Lu H, Xu M, Wu J. Stroke volume variation-guided goal-directed fluid therapy did not significantly reduce the incidence of early postoperative complications in elderly patients undergoing minimally invasive esophagectomy: a randomized controlled trial. Front Surg. 2021;8:794272.

35. Turkut N, Altun D, Canbolat N, Uzuntürk C, Şen C, Çamcı AE. Comparison of stroke volume variation-based goal-directed therapy versus standard fluid therapy in patients undergoing head and neck surgery: a randomized controlled study. Balkan Med J. 2022;39(5):351-7.

36. van Beest PA, Vos JJ, Poterman M, Kalmar AF, Scheeren TW. Tissue oxygenation as a target for goal-directed therapy in high-risk surgery: a pilot study. BMC Anesthesiol. 2014;14:122.

37. Van der Linden PL, Dierick A, Wilmin S, Bellens B, De Hert SG. A randomized controlled trial comparing an intraoperative goal-directed strategy with routine clinical practice in patients undergoing peripheral arterial surgery. Eur J Anaesthesiol. 2010;27(9):788-93.

38. de Witte P, de Witt CA, van de Minkelis JL, Boerma D, Solinger HF, Hack CE, et al. Inflammatory response and optimalisation of perioperative fluid administration during hyperthermic intraoperative intraperitoneal chemotherapy surgery. J Gastrointest Oncol. 2019;10(2):244-53.

39. Yin K, Ding J, Wu Y, Peng M. Goal-directed fluid therapy based on noninvasive cardiac output monitor reduces postoperative complications in elderly patients after gastrointestinal surgery: a randomized controlled trial. Pak J Med Sci. 2018;34(6):1320-5.

40. Zeng K, Li Y, Liang M, Gao Y, Cai H, Lin C. The influence of goal-directed fluid therapy on the prognosis of elderly patients with hypertension and gastric cancer surgery. Drug Des Devel Ther. 2014;8:2113-9.

41. Benes J, Chytra I, Altmann P, Hluchy M, Kasal E, Svitak R, et al. Intraoperative fluid optimization using stroke volume variation in high risk surgical patients: results of prospective randomized study. Crit Care. 2010;14(3):R118.

42. Hamed MA, Goda AS, Eldein RM. Comparison of goal-directed hemodynamic optimization using pulmonary artery catheter and autocalibrated arterial pressure waveform analysis Vigileo-FloTracTM system in on-pump coronary artery bypass graft surgery: a randomized controlled study. Anesth Essays Res. 2018;12(2):517-21.

43. Aaen AA, Voldby AW, Storm N, Kildsig J, Hansen EG, Zimmermann-Nielsen E, et al. Goal-directed fluid therapy in emergency abdominal surgery: a randomised multicentre trial. Br J Anaesth. 2021;127(4):521-31.

44. Cecconi M, Fasano N, Langiano N, Divella M, Costa MG, Rhodes A, et al. Goal-directed haemodynamic therapy during elective total hip arthroplasty under regional anaesthesia. Crit Care. 2011;15(3):R132.

45. Colantonio L, Claroni C, Fabrizi L, Marcelli MH, Sofra M, Giannarelli D, et al. A randomized trial of goal directed vs standard fluid therapy in cytoreductive surgery with hyperthermic intraperitoneal chemotherapy. J Gastrointest Surg. 2015;19(4):722-9.

46. Gupta P, Chaudhari SH, Nagar V, Jain D, Bansal A, Dutt A. Prospective analysis of goal-directed fluid therapy vs conventional fluid therapy in perioperative outcome of composite resections of head and neck malignancy with free tissue transfer. Indian J Anaesth. 2021;65(8):606-11.

47. Hand WR, Stoll WD, McEvoy MD, McSwain JR, Sealy CD, Skoner JM, et al. Intraoperative goal-directed hemodynamic management in free tissue transfer for head and neck cancer. Head Neck. 2016;38 Suppl 1:E1974-80.

48. Kapoor PM, Kakani M, Chowdhury U, Choudhury M, Lakshmy R, Kiran U. Early goal-directed therapy in moderate to high-risk cardiac surgery patients. Ann Card Anaesth. 2008;11(1):27-34.

49. Kapoor PM, Magoon R, Rawat R, Mehta Y. Perioperative utility of goal-directed therapy in high-risk cardiac patients undergoing coronary artery bypass grafting: "A clinical outcome and biomarker-based study". Ann Card Anaesth. 2016;19(4):638-82.

50. Kapoor PM, Magoon R, Rawat RS, Mehta Y, Taneja S, Ravi R, et al. Goal-directed therapy improves the outcome of high-risk cardiac patients undergoing off-pump coronary artery bypass. Ann Card Anaesth. 2017;20(1):83-9.

51. Kumar L, Kanneganti YS, Rajan S. Outcomes of implementation of enhanced goal directed therapy in high-risk patients undergoing abdominal surgery. Indian J Anaesth. 2015;59(4):228-33.

52. Kumar L, Rajan S, Baalachandran R. Outcomes associated with stroke volume variation versus central venous pressure guided fluid replacements during major abdominal surgery. J Anaesthesiol Clin Pharmacol. 2016;32(2):182-6.

53. Liu X, Zhang P, Liu MX, Ma JL, Wei XC, Fan D. Preoperative carbohydrate loading and intraoperative goal-directed fluid therapy for elderly patients undergoing open gastrointestinal surgery: a prospective randomized controlled trial. BMC Anesthesiol. 2021;21(1):157.

54. Martin D, Koti R, Gurusamy K, Longworth L, Singh J, Froghi F, et al. The cardiac output optimisation following liver transplant (COLT) trial: a feasibility randomised controlled trial. HPB (Oxford). 2020;22(8):1112-20.

55. Mayer J, Boldt J, Mengistu AM, Röhm KD, Suttner S. Goal-directed intraoperative therapy based on autocalibrated arterial pressure waveform analysis reduces hospital stay in high-risk surgical patients: a randomized, controlled trial. Crit Care. 2010;14(1):R18.

56. Mishra N, Rath GP, Bithal PK, Chaturvedi A, Chandra PS, Borkar SA. Effect of goal-directed intraoperative fluid therapy on duration of hospital stay and postoperative complications in patients undergoing excision of large supratentorial tumors. Neurol India. 2022;70(1):108-14.

57. Parke RL, Gilder E, Gillham MJ, Walker LJ, Bailey MJ, McGuinness SP; Fluids After Bypass Study Investigators. A multicenter, open-label, randomized controlled trial of a conservative fluid management strategy compared with usual care in participants after cardiac surgery: the fluids after bypass study. Crit Care Med. 2021;49(3):449-61.

58. Peng K, Li J, Cheng H, Ji FH. Goal-directed fluid therapy based on stroke volume variations improves fluid management and gastrointestinal perfusion in patients undergoing major orthopedic surgery. Med Princ Pract. 2014;23(5):413-20.

59. Ramsingh DS, Sanghvi C, Gamboa J, Cannesson M, Applegate RL 2nd. Outcome impact of goal directed fluid therapy during high risk abdominal surgery in low to moderate risk patients: a randomized controlled trial. J Clin Monit Comput. 2013;27(3):249-57.

60. Scheeren TW, Wiesenack C, Gerlach H, Marx G. Goal-directed intraoperative fluid therapy guided by stroke volume and its variation in high-risk surgical patients: a prospective randomized multicentre study. J Clin Monit Comput. 2013;27(3):225-33.

61. Sujatha PP, Nileshwar A, Krishna HM, Prasad SS, Prabhu M, Kamath SU. Goal-directed vs traditional approach to intraoperative fluid therapy during open major bowel surgery: is there a difference? Anesthesiol Res Pract. 2019;2019:3408940.

62. Tribuddharat S, Sathitkarnmanee T, Ngamsangsirisup K, Nongnuang K. Efficacy of Intraoperative Hemodynamic Optimization Using FloTrac/EV1000 Platform for Early Goal-Directed Therapy to Improve Postoperative Outcomes in Patients Undergoing Coronary Artery Bypass Graft with Cardiopulmonary Bypass: A Randomized Controlled Trial. Med Devices (Auckl). 2021;14:201-9.

63. Tribuddharat S, Sathitkarnmanee T, Ngamsaengsirisup K, Sornpirom S. Efficacy of early goal-directed therapy using FloTrac/EV1000 to improve postoperative outcomes in patients undergoing off-pump coronary artery bypass surgery: a randomized controlled trial. J Cardiothorac Surg. 2022;17(1):196.

64. Van der Linden PJ, Dierick A, Wilmin S, Bellens B, De Hert SG. A randomized controlled trial comparing an intraoperative goal-directed strategy with routine clinical practice in patients undergoing peripheral arterial surgery. Eur J Anaesthesiol. 2010;27(9):788-93.

65. de Waal EE, Frank M, Scheeren TW, Kaufmann T, de Korte-de Boer D, Cox B, et al. Perioperative goal-directed therapy in high-risk abdominal surgery. A multicenter randomized controlled superiority trial. J Clin Anesth. 2021;75:110506.

66. Weinberg L, Ianno D, Churilov L, Mcguigan S, Mackley L, Banting J, et al. Goal directed fluid therapy for major liver resection: A multicentre randomized controlled trial. Ann Med Surg (Lond). 2019;45:45-53.

67. Zhang J, Chen CQ, Lei XZ, Feng ZY, Zhu SM. Goal-directed fluid optimization based on stroke volume variation and cardiac index during one-lung ventilation in patients undergoing thoracoscopy lobectomy operations: a pilot study. Clinics (Sao Paulo). 2013;68(7):1065-70.

68. Zhao G, Peng P, Zhou Y, Li J, Jiang H, Shao J. The accuracy and effectiveness of goal directed fluid therapy in plateau-elderly gastrointestinal cancer patients: a prospective randomized controlled trial. Int J Clin Exp Med. 2018;11(8):8516-22.

69. Zheng H, Guo H, Ye JR, Chen L, Ma HP. Goal-directed fluid therapy in gastrointestinal surgery in older coronary heart disease patients: randomized trial. World J Surg. 2013;37(12):2820-9.
