# Supplementary material for: Goal-directed therapy guided by the FloTrac sensor in major surgery: a systematic review and meta-analysis
Source: Crit Care Sci. 2024 Apr 29;36:e20240196en. doi: 10.62675/2965-2774.20240196-en (PMC11098079; doi:10.62675/2965-2774.20240196-en)
Supplement: Translation

# Translation: Terapia guiada por metas utilizando o sensor FloTrac em cirurgia de grande porte: revisão sistemática e metanálise

**DOI:** 10.62675/2965-2774.20240196-pt

**Authors:** Márcia Regina Dias Alves, Saulo Fernandes Saturnino, Ana Beatriz Zen, Dayane Gabriele Silveira de Albuquerque, Henrique Diegoli

**Affiliations:** 1 Brasil Edwards Lifesciences - São Paulo (SP), Brasil.; 2 Brasil Universidade Federal de Minas Gerais - Belo Horizonte (MG), Brasil.; 3 Brasil Academia VBHC Educação e Consultoria - São Paulo (SP), Brasil.

**Keywords:** Objetivos, Monitorização intraoperatória, Tempo de internação, Insuficiência cardíaca, Resultado do tratamento, Unidades de terapia intensiva

## Abstract

**Objetivo:**

Fornecer informações sobre os possíveis benefícios da terapia guiada por metas utilizando o sensor FloTrac na redução de complicações pós-operatórias e na melhoria dos desfechos.

**Métodos:**

Realizamos uma revisão sistemática e uma metanálise de estudos controlados e randomizados para avaliar a terapia guiada por metas utilizando o sensor FloTrac em cirurgias de grande porte, comparando a terapia guiada por metas com os cuidados habituais ou o monitoramento invasivo em subgrupos de cirurgias cardíacas e não cardíacas. A qualidade dos artigos e das evidências foi avaliada com uma ferramenta de risco de viés e o GRADE.

**Resultados:**

Incluímos 29 estudos controlados e randomizados com 3.468 pacientes. A terapia guiada por metas reduziu significativamente a duração da internação hospitalar (diferença média de -1,43 dia; IC95% 2,07 - -0,79; I^2^ 81%), a internação na unidade de terapia intensiva (diferença média de -0,77 dia; IC95% -1,18 - -0,36; I^2^ 93%) e a ventilação mecânica (diferença média de -2,48 horas, IC95% -4,10 - -0,86; I^2^ 63%). Não houve diferença estatisticamente significativa na mortalidade, no infarto do miocárdio, na lesão renal aguda e nem na hipotensão, mas a terapia guiada por metas reduziu significativamente o risco de insuficiência cardíaca ou edema pulmonar (risco relativo de 0,46; IC95% 0,23 - 0,92; I^2^ 0%).

**Conclusão:**

A terapia guiada por metas utilizando o sensor FloTrac melhorou os desfechos clínicos e reduziu o tempo de internação no hospital e na unidade de terapia intensiva em pacientes submetidos a cirurgias de grande porte. Outras pesquisas podem validar esses resultados usando protocolos específicos e entender melhor os possíveis benefícios do FloTrac além desses desfechos.

## INTRODUÇÃO

As intervenções cirúrgicas são procedimentos médicos comuns. No entanto, apesar dos avanços nas técnicas cirúrgicas, as complicações decorrentes de um desequilíbrio entre o suprimento e a demanda de oxigênio continuam sendo causa significativa de morbidade e mortalidade.^(1)^ A hipoperfusão resultante desse desequilíbrio pode levar a uma série de complicações, incluindo lesão renal, danos a outros órgãos vitais e até mesmo à morte.^(2-4)^ O risco de complicações pode ser aumentado pelas características do paciente ou pela natureza do próprio procedimento cirúrgico. Embora a infusão de volume possa ser usada para tratar a hipoperfusão, ela não é inofensiva. A hipervolemia resultante do excesso de hidratação pode causar insuficiência cardíaca e renal, aumento do tempo de ventilação mecânica e maior tempo de internação hospitalar.^(5-7)^ Assim, o monitoramento hemodinâmico preciso é essencial para orientar o manejo de fluidos e evitar desfechos adversos.

A terapia guiada por metas (TGM) é uma abordagem abrangente e que emprega uma série de variáveis hemodinâmicas, como a variação do volume sistólico (VVS) e a variação da pressão de pulso (VPP). O objetivo é adaptar a administração de fluidos e outras intervenções terapêuticas às necessidades individuais do paciente, mantendo a perfusão adequada dos órgãos e minimizando os riscos de hipotensão e hipervolemia.^(8,9)^ As abordagens tradicionais de monitoramento hemodinâmico dependem de métodos invasivos, como medições invasivas da pressão intra-arterial, monitoramento da pressão venosa central e cateterismo da artéria pulmonar.^(10-12)^ Essas técnicas invasivas oferecem alta precisão, mas estão associadas a várias limitações, incluindo o risco de infecção, complicações decorrentes da inserção de cateteres e custos elevados.^(13-15)^ Por outro lado, os métodos minimamente invasivos podem oferecer uma alternativa mais segura, mas, muitas vezes, às custas da precisão, especialmente quando os parâmetros dinâmicos são essenciais.^(13-15)^

Nos últimos anos, os avanços nas tecnologias de monitoramento hemodinâmico minimamente invasivo, incluindo o sensor FloTrac, surgiram para preencher essa lacuna.^(16)^ O FloTrac usa um algoritmo de análise de forma de onda arterial para estimar o débito cardíaco e outros parâmetros hemodinâmicos, oferecendo uma opção minimamente invasiva promissora que supera os riscos associados aos métodos invasivos. Ele tem sido particularmente benéfico na TGM devido ao seu equilíbrio entre segurança e precisão. Entretanto, suas limitações incluem a dependência de uma forma de onda arterial estável e possíveis imprecisões em condições clínicas específicas, como arritmias.^(16)^

Metanálises anteriores sugeriram que os protocolos de TGM podem reduzir a incidência de complicações pós-operatórias, especialmente em procedimentos abdominais, ortopédicos e neurocirúrgicos importantes.^(8)^ No entanto, essas análises também destacaram uma heterogeneidade significativa nos dispositivos e protocolos usados em vários estudos, o que limitou a generalização dos resultados. Assim, é necessária uma investigação mais aprofundada para determinar os possíveis benefícios do uso do FloTrac para TGM em pacientes cirúrgicos de alto risco submetidos a cirurgias de grande porte.

Realizamos uma revisão sistemática e uma metanálise para avaliar os desfechos clínicos e o tempo de internação de pacientes em hospitais e unidades de terapia intensiva (UTIs) nos quais o FloTrac para TGM foi usado em detrimento das abordagens tradicionais de monitoramento hemodinâmico em pacientes submetidos a cirurgias de grande porte. Nosso objetivo foi fornecer informações sobre os possíveis benefícios da TGM utilizando o FloTrac na redução de complicações pós-operatórias e na melhoria dos desfechos.

## MÉTODOS

Foi realizada uma revisão sistemática seguindo as Diretrizes Brasileiras de Revisões Sistemáticas.^(17)^ Os achados foram relatados seguindo os critérios estabelecidos pela *Preferred Reporting Items for Systematic Reviews and Meta-Analyses* (PRISMA) (Tabela 1S - Material Suplementar).^(18)^

### Critérios de inclusão e exclusão

Incluímos estudos que investigaram a monitorização do débito cardíaco com FloTrac/Vigileo ou FloTrac/HemoSphere usando um protocolo de TGM em comparação com a monitorização hemodinâmica invasiva ou sem monitorização contínua do débito cardíaco (cuidados habituais [CH]) (Tabela 2S - Material suplementar). A TGM é definida como uma abordagem que usa parâmetros fisiológicos específicos para orientar as decisões de tratamento clínico. Isso pode envolver o uso de fluidos, inotrópicos ou outras intervenções baseadas em monitorização contínua ou intermitente.

Incluímos apenas estudos de pacientes adultos envolvendo cirurgia de grande porte, definida pelo Consenso Delphi entre os membros da *European Surgical Association*,^(19)^ que inclui comorbidade significativa do paciente, parâmetros cirúrgicos importantes (longa duração da operação, isquemia de órgãos, perda de sangue > 1.000 mL e muito uso de vasopressores), resposta ao estresse metabólico pós-operatório, morbidade em 30 dias > 30%, mortalidade > 2% ou necessidade de cuidados intermediários ou intensivos.

Incluímos apenas estudos controlados e randomizados (ECRs) ou revisões sistemáticas de ECRs. Os resultados comparados foram insuficiência cardíaca ou edema pulmonar (desfecho primário), lesão renal aguda, infarto do miocárdio, hipotensão, mortalidade, tempo de internação hospitalar, tempo de internação na UTI e duração da ventilação mecânica. Foram incluídos desfechos clínicos de todas as gravidades, especificamente infarto do miocárdio (biomarcadores cardíacos elevados associados a alterações compatíveis no eletrocardiograma), insuficiência cardíaca ou edema pulmonar (sinais de sobrecarga de fluido pulmonar), lesão renal aguda (redução do débito urinário ou aumento da creatinina sérica) e hipotensão (pressão arterial sistólica < 90 mmHg ou pressão arterial diastólica < 60mmHg). Estudos sem restrições de idioma foram considerados válidos para inclusão. Foram excluídos artigos publicados na forma de resumos ou cartas editoriais.

### Identificação, seleção e extração de dados de artigos

Os bancos de dados usados para pesquisar artigos foram MEDLINE, *Cochrane Central Register of Controlled Trials* (CENTRAL) e EMBASE. A estratégia de pesquisa para cada banco de dados e o número de artigos identificados são mostrados na tabela 3S (Material Suplementar), incluindo todos os artigos publicados até janeiro de 2023. Os artigos identificados nos bancos de dados foram agrupados e examinados usando o Rayyan.^(20)^

Todos os artigos foram examinados por dois revisores independentes que leram os títulos e os resumos; os artigos que foram considerados para inclusão por pelo menos um autor foram lidos na íntegra. Para a seleção final e extração de dados, os artigos foram completamente lidos por dois revisores em paralelo, e as discrepâncias foram resolvidas por meio de discussão entre os autores. O Microsoft Excel 365^®^ foi usado para registrar os dados extraídos.

### Metanálises

Para resumir os dados, a duração da internação no hospital e na UTI foi registrada em dias, e a duração da ventilação mecânica foi registrada em horas, com a conversão realizada conforme necessário. Na ausência de dados de média e desvio-padrão, convertemos a mediana e o intervalo interquartil em média e desvio-padrão, respectivamente, assumindo uma distribuição normal. Usamos a fórmula desvio-padrão = (q3 - q1)/1,35, que demonstrou ser uma abordagem superior à omissão de dados.^(21,22)^

Utilizou-se um modelo de efeitos aleatórios para a metanálise conduzida nesta revisão, para levar em conta a heterogeneidade entre os estudos incluídos. Os resultados são apresentados como razões de risco (RRs) para dados dicotômicos e diferenças médias (DMs) para dados contínuos, ambos com intervalos de confiança de 95% (IC95%). O método de Mantel-Haenszel foi empregado para estimar o tamanho do efeito combinado para variáveis binárias, e o método de variância inversa foi usado para variáveis contínuas, com o estimador de máxima verossimilhança restrito usado para a variância entre estudos (tau^2^). O método Q-Profile foi usado para calcular os intervalos de confiança para tau^2^ e tau.

Além disso, a heterogeneidade, expressa como I^2^, foi calculada usando a estatística Q (estimador DerSimonian-Laird), em que um I^2^ de 0% indica que não há evidência de heterogeneidade entre os estudos, e um I^2^ de 100% sugere que toda a variabilidade na estimativa do efeito é atribuída à heterogeneidade entre os estudos. Utilizou-se um ponto de corte de 40% para definir alta heterogeneidade. Análises adicionais de subgrupos foram realizadas para comparar os subgrupos de cirurgia cardíaca e não cardíaca e estudos com grupos de controle de pacientes com monitoramento por cateter de artéria pulmonar (CAP) e CH. O nível de significância estatística adotado foi de 5%, indicando que os resultados com valor de p < 0,05 foram considerados estatisticamente significativos. As metanálises foram realizadas com o *software* RStudio usando os pacotes *meta*, *metafor* e *forestplot*.

### Avaliação da qualidade e do viés

Avaliamos o risco de viés nos ECRs incluídos usando a ferramenta *Cochrane Risk of Bias Tool for Randomized Controlled Trials*, versão 2.0 (RoB 2.0). Cada estudo foi avaliado em sete domínios: geração de sequência aleatória, ocultação da alocação, cegamento dos participantes e da equipe, cegamento da avaliação dos desfechos, dados incompletos dos desfechos, relato seletivo e outros vieses. O estudo que recebeu um verde positivo em pelo menos três desses domínios foi considerado como tendo baixo risco de viés. O viés de publicação foi avaliado usando gráficos de funil, enquanto o teste de Egger foi aplicado para detectar possíveis assimetrias.

A qualidade da evidência foi avaliada usando a abordagem *Grading of Recommendations, Assessment, Development and Evaluations* (GRADE). O GRADE é uma ferramenta amplamente aceita, usada para avaliar a certeza das evidências em revisões sistemáticas e metanálises. A qualidade da evidência foi avaliada com base em cinco critérios: risco de viés, inconsistência, indiretividade, imprecisão e viés de publicação. A abordagem GRADE classifica a qualidade da evidência em quatro categorias - alta, moderada, baixa e muito baixa, com base na avaliação geral dos critérios.

## RESULTADOS

Inicialmente, foram identificados 855 registros por meio da pesquisa no banco de dados, com mais 12 identificados pela verificação de referências (Figura 1). Após a remoção de duplicatas, restaram 703 registros, e 634 foram excluídos com base no título e no resumo. Restaram 69 registros para análise do texto completo. Após avaliação minuciosa, 29 artigos atenderam a todos os critérios de inclusão e exclusão para a presente revisão sistemática. Os motivos da exclusão de artigos são apresentados na tabela 4S (Material Suplementar).

**Figura 1:** Fluxograma PRISMA indicando o processo de inclusão de artigos.

A presente revisão sistemática incluiu 29 ECRs realizados em vários países nos cinco continentes, com 1.733 pacientes no grupo FloTrac e 1.735 pacientes no grupo controle. Entre os estudos incluídos, 13 artigos incluíram pacientes submetidos à cirurgia abdominal de grande porte (n = 1.671), oito artigos incluíram pacientes de cirurgia cardíaca (n = 1.223), dois artigos incluíram pacientes submetidos à cirurgia de cabeça e pescoço (n = 200), dois artigos incluíram pacientes submetidos a múltiplas cirurgias (n = 184), dois artigos incluíram pacientes submetidos à cirurgia ortopédica de grande porte (n = 120), um artigo incluiu pacientes submetidos à cirurgia pulmonar (N = 60) e um artigo incluiu pacientes neurocirúrgicos (n = 40). Entre esses estudos, apenas Hamed et al.^(23)^ compararam o GDT usando FloTrac com a TGM usando uma abordagem invasiva (monitorização da artéria pulmonar), enquanto todos os outros estudos compararam a TGM usando FloTrac com CH, que não incluíam a monitorização do débito cardíaco. A tabela 1^(16,23-50)^ apresenta uma visão geral das principais características dos estudos incluídos na revisão.

**Tabela 1:** Características dos estudos incluídos na revisão sistemática

Autor	País	População
Benes et al.^(16)^	República Tcheca	Pacientes submetidos à cirurgia abdominal de grande porte de alto risco, ou pacientes com doença cardíaca isquêmica, disfunção cardíaca, doença pulmonar obstrutiva crônica moderada a grave, idade acima de 70 anos ou classificação ASA 3 ou mais
Hamed et al.^(23)^	Egito	Pacientes (entre 45 - 65 anos de idade) programados para cirurgia de enxerto de veia safena (2 ou mais enxertos)
Aaen et al.^(24)^	Dinamarca	Cirurgia de emergência para obstrução intestinal ou perfuração gastrintestinal em pacientes adultos
Cecconi et al.^(25)^	Itália	Pacientes submetidos à cirurgia de artroplastia de quadril
Colantonio et al.^(26)^	Itália	Pacientes ASA II-III com diagnóstico de carcinomatose peritoneal candidatos à peritonectomia e HIPEC
Gupta et al.^(27)^	Índia	Pacientes submetidos à ressecção de câncer de cabeça e pescoço com transferência de tecido
Hand et al.^(28)^	Estados Unidos	Pacientes adultos com cirurgia de transferência de tecido para reconstrução primária com cirurgiões oncológicos de cabeça e pescoço
Kapoor et al.^(29)^	Índia	Pacientes submetidos à cirurgia de enxerto de veia safena com EuroSCORE ≥ 3
Kapoor et al.^(30)^	Índia	Pacientes submetidos à cirurgia de enxerto de veia safena com EuroSCORE ≥ 3
Kapoor et al.^(31)^	Índia	Pacientes submetidos à cirurgia de enxerto de veia safena com EuroSCORE ≥ 3
Kumar et al.^(32)^	Índia	Pacientes submetidos a cirurgias abdominais de grande porte (aneurisma da aorta, obstrução intestinal, isquemia mesentérica, câncer de intestino envolvendo ressecção e anastomose)
Kumar et al.^(33)^	Índia	Pacientes ASA I-II submetidos a cirurgias abdominais de grande porte (procedimento de Whipple, ressecção, tumor retroperitoneal e gastrectomia)
Liu et al.^(34)^	China	Pacientes ASA II-IV com mais de 65 anos de idade submetidos à cirurgia gastrintestinal aberta (eletiva)
Martin et al.^(35)^	Reino Unido	Pacientes entre 18 - 80 anos de idade com diagnóstico de cirrose hepática e submetidos a transplante de fígado
Mayer et al.^(36)^	Alemanha	Pacientes ASA III com 2 ou mais fatores de risco submetidos à cirurgia abdominal aberta de grande porte (ressecção de intestino/estômago/fígado/esôfago, procedimento de Whipple)
Mishra et al.^(37)^	Índia	Pacientes ASA I-II entre 18 - 65 anos de idade com grandes tumores supratentoriais submetidos à craniotomia e à excisão eletivas
Parke et al.^(38)^	Nova Zelândia	Pacientes com 16 anos ou mais, classificados com EuroSCORE II de 0,9 ou mais, submetidos à cirurgia cardíaca eletiva
Peng et al.^(39)^	China	Pacientes submetidos a cirurgias ortopédicas importantes, incluindo artroplastia de quadril, fusão espinhal, fratura de fêmur e tumor sacral
Ramsingh et al.^(40)^	Estados Unidos	Pacientes submetidos a cirurgias abdominais de grande porte
Scheeren et al.^(41)^	Alemanha	Pacientes adultos ASA III-IV submetidos a cirurgias de alto risco
Sujatha et al.^(42)^	Índia	Pacientes ASA I-II com idade entre 20 - 70 anos submetidos à cirurgia intestinal eletiva
Tribuddharat et al.^(43)^	Tailândia	Pacientes adultos ASA II-IV submetidos à cirurgia de enxerto de veia safena
Tribuddharat et al.^(44)^	Tailândia	Pacientes ASA II-III submetidos à cirurgia de enxerto de veia safena
Van der Linde et al.^(45)^	Bélgica	Pacientes ASA II-III submetidos à cirurgia de revascularização do miocárdio
de Waal et al.^(46)^	Países Baixos	Pacientes submetidos à cirurgia abdominal de alto risco, como esofagectomia, duodenopancreatectomia, reparo de aneurisma abdominal, ressecção de tecidos com malignidade classificada acima de III
Weinberg et al.^(47)^	Austrália	Pacientes adultos submetidos à cirurgia de ressecção hepática eletiva de grande porte
Zhang et al.^(48)^	China	Pacientes ASA I-II entre 18 - 60 anos de idade submetidos à cirurgia de lobectomia toracoscópica sob ventilação mecânica de um pulmão
Zhao et al.^(49)^	China	Pacientes ASA II-III acima de 60 anos de idade submetidos à cirurgia de câncer abdominal de grande porte
Zheng et al.^(50)^	China	Pacientes entre 60 - 80 anos de idade com doença coronariana e submetidos à cirurgia gastrintestinal

Nos artigos científicos em que o tempo de seguimento era incerto, presumimos que os pacientes foram seguidos até a alta hospitalar. ASA - *American Society of Anesthesiologists*; HIPEC - quimioterapia intraperitoneal hipertérmica.

A avaliação da qualidade revelou que 14 dos artigos incluídos tinham baixo risco de viés, enquanto 15 tinham algumas preocupações (Figura 1 - Material Suplementar). As causas mais comuns de preocupação foram desvios das intervenções pretendidas (14 artigos) e medição do desfecho (15 artigos).

### Síntese dos resultados

A metanálise que comparou o FloTrac aos CHs não revelou diferença estatisticamente significativa entre os dois grupos em termos de mortalidade (RR de 0,97; IC95% 0,68 - 1,37; I^2^ 9%), infarto do miocárdio (RR de 0,64; IC95% 0,30 - 1,37; I^2^ 0%) ou lesão renal aguda (RR de 0,88; IC95% 0,72 - 1,07; I^2^ 0%). No entanto, o risco de insuficiência cardíaca ou edema pulmonar foi significativamente menor no grupo TGM (RR de 0,46; IC95% 0,23 - 0,92; I^2^ 0%), enquanto o risco de hipotensão não foi significativamente diferente (RR de 0,64; IC95% 0,28 - 1,45; I^2^ 42%) (Tabela 2, Figuras 2 e 3).

**Tabela 2:** Resultados das metanálises, incluindo análises de subgrupos

Desfecho - número de estudos incluídos	RR ou DM (IC95%)	Heterogeneidade (I^2^)	Teste para diferenças de subgrupo (valor de p)	Teste de viés de publicação (valor de p)
Mortalidade - 16 (RR)				
Cirurgia não cardíaca	1,01 (0,69 - 1,49)	0	0,77	0,13
Cirurgia cardíaca	0,84 (0,25 - 2,82)	0,71		
Baixo risco de viés	1,35 (0,86 - 2,10)	0	0,04	
Algumas preocupações	0,69 (0,42 - 1,11)	0		
Geral	0,97 (0,68 - 1,37)	0,09		
Infarto do miocárdio - 11 (RR)				
Cirurgia não cardíaca	0,64 (0,30 - 1,37)	0	ND	0,51
Cirurgia cardíaca	ND	ND		
Baixo risco de viés	0,55 (0,21 - 1,42)	0	0,77	
Algumas preocupações	0,72 (0,42 - 2,14)	0		
Geral	0,64 (0,30 - 1,37)	0		
Insuficiência cardíaca ou edema pulmonar - 10 (RR)				
Cirurgia não cardíaca	0,50 (0,24 - 1,02)	0	0,58	0,07
Cirurgia cardíaca	0,26 (0,03 - 2,29)	0		
Baixo risco de viés	0,51 (0,23 - 1,11)	0	0,63	
Algumas preocupações	0,32 (0,06 - 1,69)	0,17		
Geral	0,46 (0,23 - 0,92)	0		
Lesão renal aguda - 20 (RR)				
Cirurgia não cardíaca	0,87 (0,58 - 1,30)	0	0,96	0,04
Cirurgia cardíaca	0,88 (0,70 - 1,11)	0,09		
Baixo risco de viés	0,88 (0,71 - 1,10)	0	0,87	
Algumas preocupações	0,84 (0,50 - 1,42)	0		
Geral	0,88 (0,72 - 1,07)	0		
Hipotensão - 8 (RR)				
Cirurgia não cardíaca	0,64 (0,28 - 1,45)	0,42	ND	0,65
Cirurgia cardíaca	ND	ND		
Baixo risco de viés	0,46 (0,28 - 0,74)	0	0,25	
Algumas preocupações	1,17 (0,26 - 5,21)	0,56		
Geral	0,64 (0,28 - 1,45)	0,42		
Internação hospitalar - 23 (DM, dias)				
Cirurgia não cardíaca	-1,81 (-2,80 - -0,81)	0,74	0,35	0,07
Cirurgia cardíaca	-1,16 (-1,94 - -0,37)	0,89		
Baixo risco de viés	-1,19 (-2,04 - -0,34)	0,76	0,39	
Algumas preocupações	-1,79 (-2,86 - -0,73)	0,81		
Geral	-1,43 (-2,07 - -0,79)	0,81		
Internação na UTI - 18 (DM, dias)				
Cirurgia não cardíaca	-0,49 (-0,93 - -0,05)	0,73	0,21	0,22
Cirurgia cardíaca	-1,14 (-1,85 - -0,42)	0,97		
Baixo risco de viés	-0,60 (-1,17 - -0,03)	0,9	0,46	
Algumas preocupações	-0,92 (-1,53 - -0,31)	0,95		
Geral	-0,77 (-1,18 - -0,36)	0,93		
Duração da ventilação mecânica - 11 (DM, horas)				
Cirurgia não cardíaca	-3,44 (-6,13 - -0,76)	0,44	0,68	<,01
Cirurgia cardíaca	-2,19 (-4,14 - -0,25)	0,70		
Baixo risco de viés	-2,4 (-5,04 - 0,23)	0,48	0,74	
Algumas preocupações	-3,04 (-5,80 - -0,29)	0,77		
Geral	-2,48 (-4,10 - -0,86)	0,63		

RR - razão de risco; DM - diferença média; IC95% - intervalo de confiança de 95%; ND - não disponível; UTI - unidade de terapia intensiva.

**Figura 2:** Metanálises de estudos que compararam mortalidade, infarto do miocárdio e hipotensão entre pacientes que receberam terapia guiada por metas e aqueles que receberam cuidados habituais. (A) Mortalidade; (B) infarto do miocárdio; (C) hipotensão. GDT - terapia guiada por metas; RR - razão de risco; 95%CI - intervalo de confiança de 95%.

**Figura 3:** Metanálises de estudos que compararam insuficiência cardíaca ou edema pulmonar e lesão renal aguda em pacientes que receberam terapia guiada por metas *versus* aqueles que receberam cuidados habituais. (A) Insuficiência cardíaca ou edema pulmonar; (B) lesão renal aguda. GDT - terapia dirigida por metas; RR - razão de risco; 95%CI - intervalo de confiança de 95%.

Além disso, o grupo TGM apresentou internação hospitalar significativamente mais curta (DM -1,43 dia; IC95% -2,07 - -0,79; I^2^ 81%), permanência na UTI (DM -0,77 dia; IC95% -1,18 - -0,36; I^2^ 93%) e duração da ventilação mecânica (DM -2,48 horas; IC95% -4,10 - -0,86; I^2^ 68%) (Figura 4).

**Figura 4:** Metanálises de estudos comparando o tempo de internação hospitalar e na unidade de terapia intensiva de pacientes que receberam terapia guiada por metas *versus* aqueles que receberam cuidados habituais. (A) Internação hospitalar (dias); (B) internação em unidade de terapia intensiva (dias); (C) duração da ventilação mecânica (horas). GDT - terapia dirigida por metas; SD - desvio-padrão; MD - diferença média; 95%CI - intervalo de confiança de 95%.

Houve apenas um estudo que comparou o FloTrac com o CAP.^(23)^ Esse estudo relatou redução na duração do suporte respiratório (8,4 *versus* 13,4 horas; p = 0,04) e nenhuma redução estatisticamente significativa no tempo de internação na UTI (55,5 *versus* 58,1 horas; p = 0,7) ou no tempo de internação hospitalar (14,7 *versus* 16,0 dias; p = 0,6).

Após avaliar a qualidade da evidência comparando o FloTrac aos CH, os resultados revelaram evidência de baixa qualidade para lesão renal aguda e duração da ventilação mecânica e evidência de qualidade moderada para os demais desfechos (Tabela 3). A análise do gráfico de funil e o teste de Egger não mostraram sinais de viés de publicação para a maioria dos resultados, exceto para lesão renal aguda (p = 0,04) e duração da ventilação mecânica (p < 0,01) (Figura 2S - Material suplementar).

**Tabela 3:** Tabela de resumo dos resultados (GRADE)

Desfechos	Comparação ilustrativa		Efeito relativo (IC95%)	Número de participantes (estudos)	Qualidade da evidência (GRADE)
	Cuidados habituais	TGM com FloTrac			
Mortalidade	55 por 1.000	51 por 1.000	RR 0,97 (0,68 - 1,37)	2.647 (16)	Moderada*
Infarto do miocárdio	26 por 1.000	15 por 1.000	RR 0,64 (0,3 - 1,37)	1.339 (11)	Moderada *
Hipotensão	132 por 1.000	78 por 1.000	RR 0,64 (0,28 - 1,45)	514 (9)	Moderada*
Insuficiência cardíaca ou edema pulmonar	46 por 1.000	16 por 1.000	RR 0,46 (0,23 - 0,92)	1.148 (10)	Moderada*
Lesão renal aguda	119 por 1.000	99 por 1.000	RR 0,88 (0,72 - 1,07)	2.881 (20)	Baixa*†
Internação hospitalar	10,63 dias (variando entre 6 - 29)	1,43 dia (IC95% -2,07 - -0,79)	DM -1,43 (-2,07 - -0,79)	2.616 (23)	Moderada‡
Internação na UTI	2,24 dias (variando entre 1 - 7)	-0,77 dia (IC95% -1,18 - -0,36)	DM -0,77 (-1,18 - -0,36)	2.711 (18)	Moderada‡
Duração da VM	60 horas (variando entre 5 - 264 horas)	-2,48 horas (IC95% -4,10 - -0,86)	DM -2,48 (-4,10 - -0,86)	1.688 (11)	Baixa †‡

TGM - terapia dirigida por metas; IC95% - intervalo de confiança; GRADE - *Grading of Recommendations, Assessment, Development and Evaluation*; RR - razão de risco; DM - diferença média; UTI - unidade de terapia intensiva; VM - ventilação mecânica. * Imprecisão; † possibilidade de viés de publicação; ‡ inconsistência.

Além disso, quando a análise foi estratificada por subgrupos de cirurgia cardíaca e cirurgia não cardíaca, não foram encontradas diferenças estatisticamente significativas entre os dois grupos, sugerindo que o tipo de cirurgia não afetou significativamente a eficácia do FloTrac em comparação com os grupos controle. Também foi realizada uma análise de subgrupo para estratificar os estudos com base no risco de viés, categorizando-os como “baixo risco” e “algumas preocupações”. Em geral, não foram observadas diferenças significativas nos resultados entre esses grupos. A única exceção foi a mortalidade (p = 0,04), mas, apesar da diferença entre os grupos, os resultados em ambos os grupos ainda não mostraram diferença estatisticamente significativa entre o FloTrac e os CHs.

## DISCUSSÃO

A presente revisão sistemática e metanálise visou avaliar a eficácia da TGM utilizando o sensor FloTrac em comparação com as abordagens tradicionais de monitorização hemodinâmica na redução de complicações pós-operatórias e no uso de recursos em pacientes submetidos a cirurgias de grande porte. Nossos resultados sugerem que a TGM utilizando o sensor FloTrac pode levar a uma redução na incidência de insuficiência cardíaca ou edema pulmonar em comparação com os CHs (redução de risco de 54%), embora não tenha havido diferenças estatisticamente significativas em outros resultados clínicos, como mortalidade, infarto do miocárdio, lesão renal aguda ou hipotensão. Além disso, foram observadas reduções significativas na internação hospitalar (-1,43 dia), na internação na UTI (-0,77 dia) e na duração da ventilação mecânica (-2,48 horas), sugerindo que o uso de protocolos de TGM, especificamente aqueles que utilizam o sensor FloTrac, pode levar a melhores desfechos para os pacientes e ao uso mais eficiente dos recursos de saúde.

É fundamental entender que o sensor FloTrac em si não é um modificador direto dos resultados clínicos. Em vez disso, os *insights* acionáveis derivados desse sistema de monitorização hemodinâmica minimamente invasivo podem capacitar os médicos a tomarem decisões mais informadas, o que pode levar a melhores resultados para os pacientes. A orientação da TGM por meio de monitorização hemodinâmica minimamente invasiva permite que os médicos obtenham um perfil hemodinâmico mais preciso do paciente usando parâmetros dinâmicos de resposta a fluidos em comparação com os métodos tradicionais e estáticos.^(11)^ Essa abordagem permite a infusão de volume adequado, o que pode evitar os danos da hipo e hipervolemia e ajudar na tomada de decisões mais precisas em relação ao suporte inotrópico ou vasopressor.^(16)^ Os resultados deste estudo sustentam esse raciocínio, pois os protocolos da TGM utilizando o sensor FloTrac otimizaram a escolha da terapia adequada na dose correta, levando a reduções significativas na insuficiência cardíaca ou no edema pulmonar, no tempo de internação hospitalar, no tempo de internação na UTI e na duração da ventilação mecânica. Esses achados são consistentes com estudos anteriores que indicam que a manutenção da estabilidade hemodinâmica perioperatória pode reduzir a incidência de complicações pós-operatórias.^(4,51-53)^

Embora nossos dados demonstrem que o FloTrac pode contribuir para um menor tempo de internação tanto na UTI quanto no hospital, os mecanismos subjacentes não estão totalmente claros. Embora tenha sido observada uma redução significativa na insuficiência cardíaca ou no edema pulmonar, o FloTrac não foi associado a uma redução estatisticamente significativa em outros resultados pós-operatórios. Pesquisas futuras devem ter como objetivo elucidar os caminhos específicos pelos quais esses benefícios ocorrem. Também não nos concentramos em outros tipos de complicações, como infecções, que também poderiam ter um impacto nos desfechos dos pacientes e no tempo de internação.

A incorporação de dispositivos médicos na prática clínica requer uma avaliação rigorosa das evidências que respaldam seu uso. Embora existam instrumentos validados para avaliar a precisão dos métodos diagnósticos,^(54)^ a incorporação desses métodos em intervenções guiadas representa um desafio único para os dispositivos, particularmente no contexto da monitorização hemodinâmica.^(55)^ A variação nos protocolos de TGM, a curva de aprendizado dos profissionais e outros fatores podem introduzir uma variabilidade significativa em ambientes não controlados. Assim, a validação adicional de protocolos de uso específicos pode fornecer mais informações sobre a função da TGM.

Embora o presente estudo tenha sugerido que os protocolos de TGM guiados pelo FloTrac possam otimizar a escolha da terapia adequada, é importante reconhecer algumas limitações. Em primeiro lugar, não foi possível o cegamento, pois tanto os pacientes quanto os profissionais de saúde estão cientes do tipo de monitorização hemodinâmica empregada. Além disso, muitos estudos incluídos na metanálise não relataram todos os resultados buscados para as metanálises. Além disso, foram observadas diferenças significativas nas populações de pacientes e nos protocolos de TGM, aumentando a heterogeneidade da análise.

Outra limitação é a inclusão de estudos com taxa de mortalidade de 0% no grupo controle. Embora esses estudos tenham atendido a outros critérios para serem categorizados como cirurgia de grande porte, a ausência de mortes perioperatórias coloca em questão a adequação do uso da mortalidade como resultado para todas as populações cirúrgicas incluídas nesta revisão. Isso pode ser particularmente relevante para cirurgias com taxas de mortalidade inerentemente baixas, em que outros resultados, como complicações cirúrgicas ou tempo de internação no hospital ou na UTI, podem fornecer uma compreensão mais detalhada do impacto da intervenção.

No nível da revisão, as limitações incluem, entre outras, a recuperação incompleta da pesquisa identificada, a falta de dados dos participantes ou de desfechos importantes e heterogeneidade. A heterogeneidade observada em nossa metanálise pode ser atribuída a vários fatores. As populações de pacientes submetidos a cirurgias de grande porte são altamente heterogêneas, com variações nas características basais, comorbidades e procedimentos cirúrgicos, o que se correlaciona com a alta heterogeneidade encontrada na duração da internação hospitalar, duração da internação na UTI e duração da ventilação mecânica. Além disso, as variações nos protocolos de TGM, como os critérios, o tipo e o momento da administração de fluidos, podem contribuir para as diferenças nos desfechos. Outra possível limitação poderia ser a inclusão de estudos com diferentes tipos de grupos controle, ou seja, CAP e CH. No entanto, apenas um estudo empregou o CAP como controle. É interessante notar que seus desfechos não se desviaram dos achados gerais de nossa revisão.

Além disso, embora a maioria dos estudos tivesse um baixo risco de viés ou apenas algumas preocupações, muitos não forneceram definições explícitas para desfechos importantes, como infarto agudo do miocárdio, lesão renal aguda, insuficiência cardíaca, edema pulmonar e hipotensão, ou não indicaram o momento em que esses desfechos ocorreram (se intra ou pós-operatório). Essa falta de padronização pode introduzir uma camada adicional de viés na interpretação. Além disso, a experiência e o conhecimento das equipes de saúde na implementação de protocolos de TGM e na interpretação dos dados hemodinâmicos fornecidos pelo sensor FloTrac podem variar em diferentes ambientes. Essas limitações destacam a necessidade de mais pesquisas para explorar a utilidade clínica e a implementação ideal de dispositivos de monitorização hemodinâmica, como o FloTrac.

## CONCLUSÃO

Esta revisão sistemática e metanálise sugeriu que os protocolos de terapia guiada por metas utilizando o sensor FloTrac levam a melhores desfechos clínicos e reduzem a internação hospitalar e unidade de terapia intensiva e o tempo de ventilação mecânica em pacientes submetidos a cirurgias de grande porte. São necessárias mais pesquisas para validar os resultados deste estudo para protocolos de uso específicos e para entender melhor os possíveis benefícios do sensor FloTrac, além dos desfechos medidos nesta revisão. Apesar dessas limitações, o presente estudo fornece informações importantes sobre os possíveis benefícios da incorporação da monitorização hemodinâmica minimamente invasiva na prática clínica e destaca a necessidade de pesquisas contínuas nessa área.
